# ‘Everything takes too long and nobody is listening’: Developing theory to understand the impact of advice on stress and the ability to cope

**DOI:** 10.1371/journal.pone.0231014

**Published:** 2020-04-23

**Authors:** Jawwad Mustafa, Philip Hodgson, Monique Lhussier, Natalie Forster, Susan Mary Carr, Sonia Michelle Dalkin

**Affiliations:** 1 Faculty of Health and Life Sciences, Northumbria University, Newcastle, United Kingdom; 2 Fuse (The Centre for Translational Research in Public Health), Newcastle, United Kingdom; University of Indianapolis, UNITED STATES

## Abstract

Shrinking state spending in the UK has been accompanied by a profound restructuring of the welfare system, leading to financial insecurity for many people, culminating in extreme stress and serious deterioration of physical and mental health. Theory surrounding the impact of welfare advice on stress is lacking; this paper undertakes an in depth exploration of the experiences of stress among welfare advice seekers, considering these in light of existing substantive theories of stress and coping to generate new insight. A thematic analysis explored the experiences of stress in welfare advice seekers. Four overarching themes and twelve subthemes emerged. They are further understood utilising traditional theories of stress (Transactional Model of Stress and Coping and the Conservation of Resources theory), which then underpin the development of a ‘Stress Support Matrix’ and a holistic theory related specifically to welfare, stress and coping.

## Introduction

### UK welfare context

Social welfare benefits are hypothesised to improve health-related quality of life, mediated by reduced stress, the adoption of more advantageous social arrangements and healthier behaviours [1]. Yet since 2010, successive governments in the UK have embarked on a programme of austerity, involving cuts to the funding of public services, [2,3] including social welfare benefits. This constitutes the most significant and longest-sustained reduction in state spending since World War II [4]. The Department for Work and Pensions (DWP), which is responsible for welfare payments has seen an unprecedented level of cuts, with a 35% reduction in funding in the period from 2010 to 2015 [5]. Public sector budget cuts are to continue until 2020, with the DWP remaining a key target [6].

Alongside this sustained period of shrinking state spending in the UK [7] the welfare system has also been profoundly restructured [8]. Changes have included replacing the Disability Living Allowance (DLA) and Incapacity Benefit with Personal Independence Payment (PIP) and Employment Support Allowance (ESA) respectively, introducing a benefits cap and assessing claimants with an overhauled Work Capability Assessment to determine whether they are ‘fit-for-work’ [8,9]. These changes have led to financial insecurity for many people [10]. The Work Capability Assessments have been deemed culpable for many welfare recipients being wrongly deemed fit for work and have led to serious consequences, including extreme stress and serious deterioration of physical and mental health [11]. The ensuing culture of punitive sanctions through which benefits are taken away from those who do not fulfil the conditions of welfare settlements has worsened the prospects of claimants returning to work [12]. This in turn leads to more undesirable financial, health and behavioural outcomes, including increased stress [13,14]. In light of these complex and potentially multiple health consequences of welfare-related stress, this paper undertakes an in depth exploration of the experiences of stress among welfare advice seekers, considering these in light of existing substantive theory on stress and coping. The main theories to be considered are the Transactional Model of Stress and Coping [15] and the Conservation of Resources theory [16].

### Stress: Definition and theory

Stress is a complex concept [17], which lacks a consensual definition and a universally accepted framework [18–20]. For the purposes of this study, we adopted Lazarus’ [21] definition of stress as arising: “when individuals perceive that they cannot adequately cope with the demands being made on them or with threats to their well-being”. This definition meets the dual aim of acknowledging the individual experience of stress and also the importance of the wider contextual conditions.

Understanding both the individual aspect of stress and wider factors is key to comprehending stress experiences. Lazarus & Folkman [18], in the Transactional Model of Stress and Coping (TMSC) defined stress as “a product of a transaction between a person (including multiple systems: cognitive, physiological, affective, psychological, neurological) and his or her complex environment” [18]. It allows the individual to determine what is stressful to them and whether they have the ability to respond to the stress while taking into consideration the person’s environment. This appraisal is explained as two separate, simultaneously-occurring, processes [22]. The primary appraisal is identifying the type of stress that is incurred; whether it is relevant or not, whether it is stressful or not, whether it is a harmful threat or a positive challenge [22]. The secondary appraisal is assessing whether they have the necessary social, physical, psychological and material resources to overcome the threat [23,24]. As an extension to the TMSC, Hobfoll [16,25] explains that objective resource evaluation is also central to stress appraisal and understanding. Grandey and Cropanzano [26] explained the theory by defining stress as ‘a reaction to an environment in which there is the threat of a loss of resources, an actual loss in resources, or lack of an expected gain in resources’ (pg.352). Being able to apply stress theories to real-world phenomena is the litmus test in seeing whether they help to give an accurate insight into stress.

Building theories is important in understanding social and psychological phenomena, but there is an evident need to go beyond the abstract hypotheses that make up theories to ensure that they can be applied to the real world [27]. A particular criticism of stress and coping research is that there is a lack of applied research that puts theory into practice [28]. As a theory constructs a framework for explaining behaviour and behaviour change [29], applying these theories to stress experiences would allow for an understanding of both the personal and environmental elements of the stress process. By looking at organisations that act as resources for people in times of stress, we can apply these theories and ascertain their validity and explanatory potential. Citizens Advice (CA) in the UK is an example of such a service.

### Welfare advice service setting

CA describe their service as ‘free, independent, confidential and impartial advice to everyone on their rights and responsibilities’ [30]. Although it provides a range of services, a lot of the work that the service does is helping clients with welfare related issues. In the period of October 2017 to September 2018, the service as a whole provided 4,141,044 consultations, 55% of which related to state benefits, Universal Credit and debt [31]. Recent research has found that a CA service had a positive impact on its clients through increasing wellbeing and reducing stress [32]. This paper delves further into this experience of stress, and stress relief through the service, concurrently developing further understanding in stress theory and research.

### Background to the study

This article reports on a secondary data analysis. The original study was a realist evaluation of a CA service in the North East England [32,33]. It is pertinent to look at this geographical location, as it is one of the areas most prominently affected by cuts in the UK [34]. Data from the study included qualitative interviews with clients in receipt of CA support. Whilst the initial focus was to elucidate how, why and in what circumstances the service worked best, one key finding was that there was a marked improvement in wellbeing and reduction in stress for welfare advice seekers after accessing the service [32,33]. Currently, theory surrounding stress, ability to cope and advice is sparse. In order to develop a systematic way of understanding this interaction and to progress research in this contemporary area, it is essential that both relevant data and current theoretical models be considered to develop further theory and enhance understanding.

### Research question and aims

The research question for the secondary analysis reported here is ‘What are the experiences of stress for welfare advice seekers before and after receiving support from a Citizens Advice service?’ The objectives of the project are to understand the nature of the stress experienced, how it was described by participants and the mechanisms through which it was reduced.

## Methods

As the primary study revealed that there had been a reduction in stress for those that had accessed advice services when dealing with welfare issues, it was thought to be important to take a full and insightful account of their experiences in order to account for what enabled an improvement in wellbeing. While individual perception plays a big part in stress, bringing personal insights together and understanding them as a collective to identify patterns could help in understanding how stress manifests and what can be done to help on a holistic level. The project was given approval through the Northumbria University Ethical Approval system (reference: HLS-PHW1411524).

### Participants

A purposive sample of clients (n = 22) attending CA for welfare support were recruited to the study via telephone. They had all attended one of three more intensive advice service, where the claimant’s issues are resolved in the course of a number of meetings. Interviews took place between March and October 2016 and lasted between 30 minutes and an hour. Interviews took place at the participant’s home or Citizens Advice. All participants approached took part in the study. Data saturation was reached.

### Data capture

The interview data was collected using a semi structured interview schedule; data was collected by three experienced academic researchers (SMD, NF, PH; SD and PH PhD, NF PGCert) all of whom were employed by Northumbria University as researchers. The interview questions were broad and included examination of multiple and unique aspects of stress. This meant that the data collected was expansive and thereby suitable for secondary analysis [35–37]. Data was audio recorded and transcribed verbatim and field notes were taken.

### Data analysis

Data was analysed by JM using NVivo, following the six steps of thematic analysis as explained by Braun and Clarke [38]. It was noted that these steps were not linear but rather contributed to a loose and recursive process. The process is detailed below.

1. Familiarisation: The transcripts and audio recordings were used for data immersion for the purposes of this analysis. The interviews were listened to at least once each and the transcripts were read multiple times to build a clear insight into what was being communicated [38]. During these familiarisation processes, notes were made of common words and initial ideas, guided by, but not restricted, to the stress theories considered here.
2. Creation of codes: The transcripts were then uploaded on to ‘Nvivo 12’ to allow easy creation of codes. The researchers used the initial notes to create a base for the codes and re-read transcripts to ensure other pertinent points had not been missed. By the end of the coding process, 75 codes and sub-codes were created, which were merged and deleted where appropriate, to answer the research question and informed by the substantive stress theories identified as relevant from the literature.
3. Theme building: Codes were grouped under larger themes which took the form of a timeline, from initial interaction with stress to after effects of advice. An inductive approach to thematic analysis was used alongside the theoretical approach. Themes first helped in terms of building insight into stress perception and influencing factors, while the theoretical approach aided in identifying the stress reaction and subsequent coping processes.
4. Reviewing the themes: This process was informed by Patton [39]’s criteria for judging categories of ‘internal homogeneity’ and ‘external heterogeneity’. After revising the themes, the names and definitions of themes were revisited to ensure that they reflected the content [38].
5. Naming the themes: The four overarching themes created were: 1) Initial Interaction with Stress, 2) Coping with Stress, 3) Intervention from Citizens Advice, 4) After-effects of Advice (Table 1).
6. The sixth and final step included putting together all the themes and subthemes and selecting key extracts that illustrated them. This involved crafting a credible narrative that answered the research question and through which the reader could understand the validity and relevance of the findings.

**Table 1:** List of themes and subthemes.

Theme	Subtheme
Initial Interaction with Stress	Causes Personal outlook
Coping with Stress	Appraisal and resource identification Coping behaviours Support networks Stigma
Intervention from Citizens Advice	Staff Perception of Citizens Advice Resource Gains
After-effects of Advice	Support System Changes Improved Quality of Life

Consolidated criteria for reporting qualitative research (COREQ) is adhered to and provided as a supplementary file.

## Findings

The data is presented in four overarching themes (see Table 1). Presenting the data in this way allows for a linear and thorough inspection of stress experiences, how they were relieved and the lasting impact of the interventions. Twelve subthemes emerged, which elaborate on facets of the overarching narrative.

### Initial Interaction with Stress (Theme 1)

This theme explores the individual aspects of CA clients’ experience of stress by exploring its root causes, the subjective perception of their situation and how their unique life experiences influence this.

#### Causes

The primary sources of stress for advice seekers were related to welfare support. These issues included but were not limited to: housing benefit, Employment Support Allowance and Personal Independence Payment. Many of the participants had come to CA to receive assistance in dealing with welfare changes, for example switching from an old benefit to a new one, or for help with appeals for decisions deemed unfair or incorrect.

The recent and complex changes made to the benefits system [8,9] were a cause of stress itself, making participants apprehensive, with their primary concern being the risk of losing their financial support:

*“I’ve also got to worry about my disability benefit cause that’s got to get changed to PIP and I’m waiting to hear from that cause I only get the low rate of that so that might be getting stopped altogether for me so that’s another concern as well because if that stops I don’t know if I’m going to be able to cope on that other money*”

(Participant 26)

There was also a real fear that the loss of financial support would lead to further loss in the near future, including a loss of secure housing:

*“I would have ended up losing my benefit*, *I would end up losing me flat and everything you know what I mean*”

(Participant 22)

In some cases, the stress arising from welfare issues was exacerbated by other life circumstances such as grief and debt accumulation. These multiple and complex situations increased the stress levels occurring from welfare changes and potential loss of benefits. Furthermore, participants stated that additional stress was caused by institutions such as the Department for Work and Pensions and the Jobcentre, which were described as:

*“Uncaring*, *they’re so abrupt and rude*”

(Participant 9)

The environment created by government organisations was perceived as hostile and unwelcoming:

*“Oh I don’t like the jobcentre*, *that’s like going in a piranha tank*.”

(Participant 26)

This feeling of hostility intensified the stress experienced by participants, as they felt that the Jobcentre was not supportive and only existed to take away their entitlements. As Participant 5 notes, they’re simply *“looking at ways of kicking people off benefits”*. Participant 22 provides further support to this point: *“because they don’t want people like me to have access and help”*. The distrust of governmental organisations added to feelings of hostility and helplessness, where participants saw the Jobcentre as an accusatorial and watchful tyrant waiting to catch them out: “*They’re all mini Hitlers”* (Participant 3)

Waiting times for decisions regarding the outcomes of claims added to this stress:

*“It’s just a waiting game but it is stressing me out*”

(Participant 10)

Prolonged stress was experienced when further delays to payments occurred, after appeals had been won.

#### Personal outlook

The way that stress is initially seen is dependent on the personal outlook of the individual. This is determined by the distinctive life experiences that a person learns from the early developmental stage through to adulthood and continuing to old age [40,41]. Past experiences with stress usually shape how a person deals with situations that put pressure on the individual [42]. Among the participants there were those that had poor experiences in dealing with stress from childhood:

*“It follows on from a lot of my problems since childhood* (…) *I was in social care when I was small*.”

(Participant 10)

Participants who previously experienced difficult life circumstances, may have experienced more intense stress responses, or may have felt ill equipped to deal with stress. The Adverse Childhood Experiences (ACE) study found that adults that faced developmental adversity and toxic stress as children were more likely to suffer a higher risk of chronic disease later in life, leading to adverse impacts on the immune, nervous and cardiovascular systems [43], resulting in a flawed stress schema. The stress caused by welfare issues often served to exacerbate pre-existing conditions:

*“Well*, *put it this way*, *before I was ill I was an avid mountain climber*. *I used to fell walk*. *I used to fish a lot*. *I used to weight train*. *Training quite a bit—you know*, *quite a lot*. *And now I can’t do anything at all*.”

(Participant 20)

The hopelessness resulting from inactivity also increased a sense of helplessness as participants had a general feeling that they were unable to improve their material situation. This situation could become self-fulfilling; as one is unable to overcome stressors, they then felt less equipped to deal with new ones [44]. Confidence is a key in the belief that stress can be alleviated:

*“I thought I do not want to fill this form in wrong and screw it up for myself*”

(Participant 9)

Confidence in one’s ability also included whether one has the required tools to relieve stress:

*“I felt at first inadequate because I thought well*, *I should be able to fill a form in*”

(Participant 7)

The feeling of inadequacy is pertinent as it demonstrates participants’ inability to deal with the primary factor that is causing their stress. It would also limit their sense of self-assurance and then lead to feelings of hopelessness and failure. The combination of feeling inadequate and lacking control further worsens the stress that the participants feel and subsequently impedes their belief in their ability to change their situation.

### Coping with Stress (Theme 2)

This theme examines participants’ positive and negative behaviours in response to stress, as well as an exploration of the dynamics of social and practical support relevant to welfare stress.

#### Appraisal and resource identification

Reactions to stress were based on an assessment of what resources participants had available to them to manage their stress. One of the main organisations that the participants were directed to was the Jobcentre. However, rather than assisting with diminishing stress, the Jobcentre both caused and enhanced stress. Participants highlighted that when the Jobcentre did provide support, it often conflicted with other sources, confusing and too complicated to understand:

*“I went to the jobseekers signed us off to go to work and the other (GP) one’s saying I’m not fit enough to go to work so I cannot understand them*, *one’s contradicting the other one you see*”

(Participant 15)

When providing assistance, the staff at the Jobcentre seemed to be apathetic, unconcerned and insensitive about the concerns of the participants:

*“[Jobcentre staff] don’t comprehend what happens in the real world*. *You know*, *where they’ve got no concept of [circumstances]*”

(Participant 24)

When looking at the Jobcentre as a resource, participants did not feel that they helped with the stress causing concerns:

“Everything takes too long and nobody is listening.”

(Participant 18)

When the resource offered does not feel like an adequate point of assistance, then it is unlikely to reduce stress.

#### Support networks

Having support systems in place helped to cushion the stress experienced by participants. It meant that participants had people around them that they could feel emotionally supported by while there was insecurity about their material status:

“Stressed ah if I get like down I’ll phone my mam, just like before I spoke to her and I went ‘Mam can I come down for an hour (…) I’ve got aye I’ve got all my family”

(Participant 11)

However, there was a deficiency in practical support, for assistance with forms filling for example. This led to a continuation of stress, as claimants could not resolve their problems directly through their support networks:

*“Yeah I’ve got plenty of back up from family friends but it’s back up from somebody who knows the system and that’s the big difference*, *because as a lay person you don’t know the system*.”

(Participant 24)

Where there was a lack of both emotional and practical support, this fuelled feelings of isolation and stress:

*“Who else do we turn to*? *Like we didn’t… Like*, *[Partner] hasn’t really got any family*.”

(Participant 14)

Those that had emotional support but lacked practical help made up a large proportion of the study, the absence of practical support resulted in the participants resorting to a variety of coping behaviours as they could not resolve their situation immediately.

#### Coping behaviours

When participants experienced stress, they adopted coping behaviours that acted as outlets for their stress. These outlets were temporary or long-term and either helped or hindered the management of stress. Many of the participants showed a propensity to evade their problems through emotion-oriented behaviours, such as procrastination and avoidance:

*“Tend to procrastinate and put things off*”

(Participant 2)

*“I used to put it on a table in a pile*, *basically*. *It’s like bills—you know you can’t pay them*, *so you just throw them*”

(Participant 18)

These behaviours resulted from the previously mentioned lack of confidence, control and belief in ability to change. It is these behaviours that extend the period of stress as the problems were not being dealt with [45]. Emotion-oriented behaviours also manifested in unhealthy activities that distracted the participant from their stress, these included smoking cigarettes, drinking alcohol and taking drugs:

*“I smoke a hell of a lot when I’m stressed*, *a hell of a lot more you know*, *as I say I was on 800 fags a you know it’s a lot of [cigarettes]*”

(Participant 12)

*“I mean I’ve*, *I drink alcohol and take drugs*”

(Participant 22)

Unhealthy behaviours were also expressed in unproductive ways that had the potential to alienate limited sources of social support:

*“I get agitated*, *snappy*, *angry*. *I swear a lot and shout*.”

(Participant 14)

However, among the participants were also those that used healthy behaviours to cope:

*“I’ll go out for a day out*.”

(Participant 5)

Other participants showed a problem-solving attitude to their stress. They possessed a mindset that made them want to resolve the causes of stress so that they could relieve themselves of the insecurity that they were feeling:

*“You just get on with it*, *I suppose*.”

(Participant 17)

It is possible to say that even those that exhibited mainly emotion-orientated behaviours possessed a degree of problem-solving attitudes as all participants accessed CA. Thus, participants moved beyond their emotion-oriented behaviour to problem-oriented behaviour, though this would typically come after a period of stress.

#### Stigma

Participants’ opinions about welfare and social security affects how they view the stress that arises from needing welfare support themselves. The different sources of welfare stigma [46–48] played a role in this, but social stigma was the biggest concern:

*“Yeah*. *More to do with that and having self-respect basically*. *Because it’s all these programmes and that*. *Being on benefits is all*, *frowned upon*.”

(Participant 3)

The social stigma attached to receiving welfare made participants feel like they should not apply for welfare support:

*“Cos I felt like ‘oh my god’ because I’ve always worked*, *and I felt like a scrounger*.”

(Participant 3)

These thoughts not only increased stress but also had an impact on the uptake of assistance that would have helped them:

*“Because people are embarrassed*, *they don’t want to come and ask for help*”

(Participant 1)

The non-uptake of support because of the fear of stigma can prolong the duration of stress experienced. In addition to social stigma, there is a feeling that welfare recipients are stigmatised by the government too:

*“You see the negative thing always came from that you wouldn’t get anything off the government*”

(Participant 23)

However, this was mitigated by social factors:

*“That’s one thing my dad always says*, *there’s no shame in asking for help*.”

(Participant 26)

This discrepancy could be explained by the predominance of two groups; those who had been on welfare benefits on a long-term basis and those that had only recently needed welfare support. Those that had more experience with the welfare system did not feel as though stigma impacted their perception as much as they believed that they were in need of it and entitled to it. The newer claimants were either in paid employment or had been until recently, and were worried that they should not be seeking support due to the current stigmatisation of ‘benefit scroungers’ seen in the media [49].

### Intervention from Citizens Advice (Theme 3)

This theme comprehensively examines which features of CA were helpful in relieving stress and conversely which parts were less impactful.

#### Perception of Citizens Advice

Participants described being unsure of the primary function of CA; they were unsure of how the service worked and whether a referral was necessary in order to gain access to the service:

*“I just didn’t know how easily accessible it was*. *I thought it was gunna be like*, *well at one*, *I didn’t know where they were and two*, *I just thought you*, *I didn’t know you could just come in and do the numbers system*. *(…) And I didn’t realise how much they actually done*.”

(Participant 3)

The lack of awareness surrounding the service meant that participants were mainly accessing it through recommendations or referrals as opposed to self-referral. This signified that those with limited networks were less likely to know about the service and its function, and therefore were less likely to access it. Once the participants were aware of CA, most were positive about seeking support from the organisation:

*“I’ve got nothing to lose really*.”

(Participant 15)

Participants indicated that they did not contact CA as early as they could have. This could be a consequence of their interactions with the Jobcentre and further stimulated by the perception that even if they did seek support, they would be unlikely to get any help, financial or other:

*“I’ve heard people on about it and that*, *I think that was partially why I never went*. *If you know what I mean because*, *you know when you*, *sort of even when you’re working*, *you think*, *ah you can’t claim anything*”

(Participant 23)

There was a general feeling that participants had benefited from accessing CA as they received the support that they needed:

*“Yeah*, *yeah*. *I think*, *you know*, *ultimately the fact that the appeal […] was successful […] I don’t think it would have been successful without her help*. *And if it wasn’t successful then that stress would have continued*.”

(Participant 17)

#### Advisors

Participants were extremely positive about the experience that they had with the advisors at CA. They stated that they had built a lasting relationship with the them, which was facilitated by the advisor’s desire to build rapport and their expertise in doing so. Participants commented on the value of this:

*“Well the second tribunal [advisor] supported him and said*, *‘I’ll be there’ and he went*, *he wouldn’t of went full- he just absolutely couldn’t of went*, *but having that friendly familiar face* …”

(Participant 13)

The advisors would treat each client as an individual and show equal care and concern to their case. This was different from the treatment that they received at the Jobcentre where they perceived the service to be impersonal. There was a perception that advisors were committed to providing an empathetic service:

*“My situation was understood*, *considered*”

(Participant 9)

*“They talk to you as a person*, *not as just one of the masses*.”

(Participant 18)

Advisors would remember the participants, so they did not need to recount their situation every time they came to CA, this also enabled participants to feel like they had a specific point of reference:

*“And I just… She knew more about me than I did*. *It seemed that way*, *you know*. *So because she knew and took the time to remember stuff like that*, *it just made it feel more comfortable*.”

(Participant 2)

Advisors showed a level of care and interest that enabled a trusting relationship to develop. This allowed participants to let their guard down, overcoming the prior suspicion that they had built up from experiences with the Department for Work and Pensions and Jobcentre. This is supported by participants positively referring to the independence and impartiality of CA compared to the Department for Work and Pensions and the Jobcentre:

*“Yeah*, *yeah*, *they’re focused on you*. *You know*, *they haven’t got the interests of*, *you know… You know*, *for instance*, *the DWP or the council or*, *you know*, *anybody like that*”

(Participant 17)

The level of knowledge and expertise of the advisors was another major factor that participants felt was integral to the service:

*“She just seemed to know all the little tricks of the departments if you like*”

(Participant 2)

The rapport that was built with the participants resulted in them feeling comfortable sharing their issues and seeking help; participants came to count the CA advisor as part of their social support network:

*“It’s like talking to*, *like*, *a family member or a friend*, *isn’t it*, *like*, *that you’ve got*”

(Participant 14)

#### Resource gains

Accessing CA gave the participants a resource to rely on to relieve stress. They considered the organisation as dependable and having the ability to assist with their issues. Thus, once support from CA was in place, the participants were able to reassess their situation and felt more at ease, even when their issues were not fully resolved:

Participant 25: “*Knowing that I’ve got the help there*, *[I can cope better] yeah*.”

After accessing CA, clients were able to access more specialist services that could help them further. The participants were referred to a range of services such as debt advice or specific support groups:

*“And they put me on to somebody and they took a Debt Relief Order out for me*. *I think that’s what you call them*. *Debt Relief Order or something*. *And they sorted that out for me*. *But also my benefits*. *They helped me to apply for my benefits as well*.”

(Participant 25)

The advisors also focused on improving health outcomes holistically by working to help participants get access to services that would allow them to not only reduce stress but also enhance their general wellbeing:

*“So she got me mobility car which is the most important er thing of the lot*”

(Participant 5)

### After-effects of Advice (Theme 4)

This theme looks at how advice changed participants’ lives generally but also how it affected their stress processes. It shows changes to their perception of stress and crucially how they would manage future stress.

#### Improved quality of life

Participants demonstrated improvements as a result of receiving advice. These varied from better physical health outcomes to enhanced wellbeing and positivity along with a reduction in stress. Participants were grateful for the support that they had received from CA as it enabled them to resolve their welfare issues and, in some cases, other issues that were related to their wellbeing. The key to the relief of stress was widely attributed to the CA intervention:

*“I’m relieved*, *because I don’t know what I would do if I didn’t have them (Citizens Advice) for advice*.”

(Participant 25)

Participants reported wide ranging benefits after receiving support, notably, they mentioned increased confidence, reduced insecurity and most importantly reduced stress:

*“But now*, *with [Advisor] and [Name]*, *I’m feeling more confident*. *As I said earlier*, *I’m getting my mojo back and I can go and do things*.”

(Participant 2)

*“Just totally stress free*.”

(Participant 3)

Positive health benefits were also reported by participants. Although complex issues are never addressed in isolation, a reduction in stress could have contributed to participants’ self-efficacy for change:

*“I actually packed in smoking like I say*, *err*, *the wife’s cut down a lot*, *and we’re doing more exercise*.”

(Participant 23)

However, it should also be noted that these effects were not often reported from participants with long-term health conditions. Even when they had benefited from the advice, it only had a limited effect on their wellbeing as they had other existing problems that CA could not directly help with:

*“When I get the money it’ll be it’s there but it’s it’s me illness that stops is not the money do you know what I mean*, *I mean if I could have a million pound in the bank I still wouldn’t be able to do the things that you can do*”

(Participant 11)

#### Changes

Along with an improved quality of life, participants also showed changes in behaviour and perspective. There was a noticeable difference in the way that participants had dealt with stress. While many had initially focused on emotion-oriented coping, there was a clear move towards use of problem-solving based behaviours after receiving advice:

*“So instead of procrastinate*, *I know I’ve got to go and sort something out*.”

(Participant 2)

There was also a recognition of how to deal with stress in a positive manner:

*“I recognise that I’m stressed now*, *and I’m kinder to myself now than I used to be*. *I used to just plough on and keep going and I wouldn’t see*.”

(Participant 9)

Participants also noted feelings of control once they had accessed the service.

*“Yeah I did like [feel more in control] once things started to get a bit easier*”

(Participant 1)

#### Support system

Many of the participants had adopted CA as a future reference point for their welfare issues, or even as part of their permanent support system:

*“Last year I went to Citizens’ Advice and [advisor] took me on*. *And*, *she’s still supporting (…) I won’t stop using them*”

(Participant 10)

For some, CA acted as a form of both social and practical support, there was evidence that this continued to prevent these participants from feeling isolated:

*“Yeah I feel a lot happier knowing that there’s somebody there that can actually help erm because my parents and me we don’t really get along that well*”

(Participant 26)

While participants felt able to go back to CA for future advice and support, it was clear that the service fostered this relationship and reassured participants that they could do so:

*“Well it’s been about*, *mmm*, *I’m trying to think about four years now since we first met [advisor] and she done the first lot of forms for the DLA and he got awarded it indefinitely but then they changed it to PIP and I thought ‘ah God this is where it rocks the boat I’m gonna phone the CAB and I’m gonna get’ but she can’t*”

(Participant 13)

## Discussion

### Summary of findings

The findings indicate that participants were struggling with issues relating to welfare advice, mainly with the bureaucracy of the changes and appeals to the decisions that were made on their claim. This stress was further embedded by the lack of support received from the Jobcentres and the DWP. Individual situations and stress schemas played either a mitigating or an aggravating part in how stress was received and dealt with. Coping behaviours, mainly negative in the case of welfare advice seekers, initialised stress reactions. They showed improvement once accessing CA, and they attributed their progress to the organisation’s staff and the accessibility of the service. This was also shown to have had a subsequent positive impact on their wellbeing.

### Theory application

The main theories that were considered relevant to the data were the Transactional Model of Stress and Coping [18] and the Conservation of Resources theory [16]. Both of these theories consider not only the individual’s biological, physical and psychological experiences of stress, but the wider social impacts of their stress and coping mechanisms.

The Transactional Model of Stress and Coping [15] provides an explanation of the stress experienced by participants. A primary appraisal led participants to decide whether the threat (possibility of welfare support loss) was relevant and harmful or not. A secondary appraisal allowed them to consider whether they had the capacity to deal with the stress. Lazarus and Folkman [18] describe how individuals make a judgement on whether they believe they have the necessary social, physical, psychological and material resources in order to surmount the stress. Participants to this study mentioned mainly social and material aspects of coping.

The Conservation of Resources theory by Hobfoll [16] elucidates this process in detail. Claimants evaluated the resources that they had at their disposal to enable them to deal with stressful situations. When considering the resources, they looked at whether they had the personal competence, social support and practical assistance to enable them to deal with their stress. The personal competence aspect included individual qualities to deal with stress, including hardiness [50], locus of control [51], self-efficacy [52] and sense of coherence [53]. Many claimants had social support but lacked practical assistance to deal with the welfare systems requirements.

Two further key principles of the theory were manifest within the data. The first principle was the primacy of resource loss, which saw participants experience greater stress at the threat of lost resources than could simply be regained by replacing those losses. The principle states that the loss would be felt more intensely and acutely than gain. This is most evident from the reaction of those participants who had long-term health conditions and did not feel that accessing services had a major impact on their wellbeing. The principle further asserts that a loss would lead to further loss and gains would lead to further gains [54]. Participants had a clear worry that the loss of the financial support would lead to further losses in their circumstances, this was highlighted in fears of losing food, housing and financial means for physical exercise. Meanwhile there were clear signs of participants making further gains from the initial gain that they made in the form of CA. This was seen in procurement of or referrals to organisations that dealt with debt relief, provided physical support apparatus and also support groups.

The second principle of resource investment looks at how people invest in resources to protect against resource loss, recover from losses and gain resources [25]. Participants demonstrated this by appealing the decision that was made to take away their welfare support to prevent loss. They also did so by looking for resources, such as CA, that would aid them in mitigating the stress that they were feeling. The principle further considers how individuals look at future protection by investing in resources. Participants were positive that they could use CA as a resource and asserted that they would use it in the future. This is reinforced by the Proactive Coping theory proposed by Aspinwall and Taylor [55], wherein it is described that participants look for resources to help them with future stress.

In analysing how the CA resource was effective, it is understood that the main aspect was the supportive environment fostered by the friendly, empathetic and helpful staff at the service. Zimmer-Gembeck and Locke [56] highlighted that when positive and supportive relationships are fostered, people demonstrate more active-coping behaviours. This was the nature of the relationship that participants had with CA.

### Applications

#### Support

Among the applications of the study is the understanding of the nature of support. Here support is looked at as two different branches; emotional support and practical support. Emotional support has been found to encourage positive wellbeing [57,58], however, recent analyses have shown that this is multifaceted and depends on context [59,60]. In this study, emotional support was found to be beneficial to the participants during their time of hardship. Although it did not relieve participants of their stress completely, it buffered the emotional impacts of stress [57,61–63].

**Fig 1:**
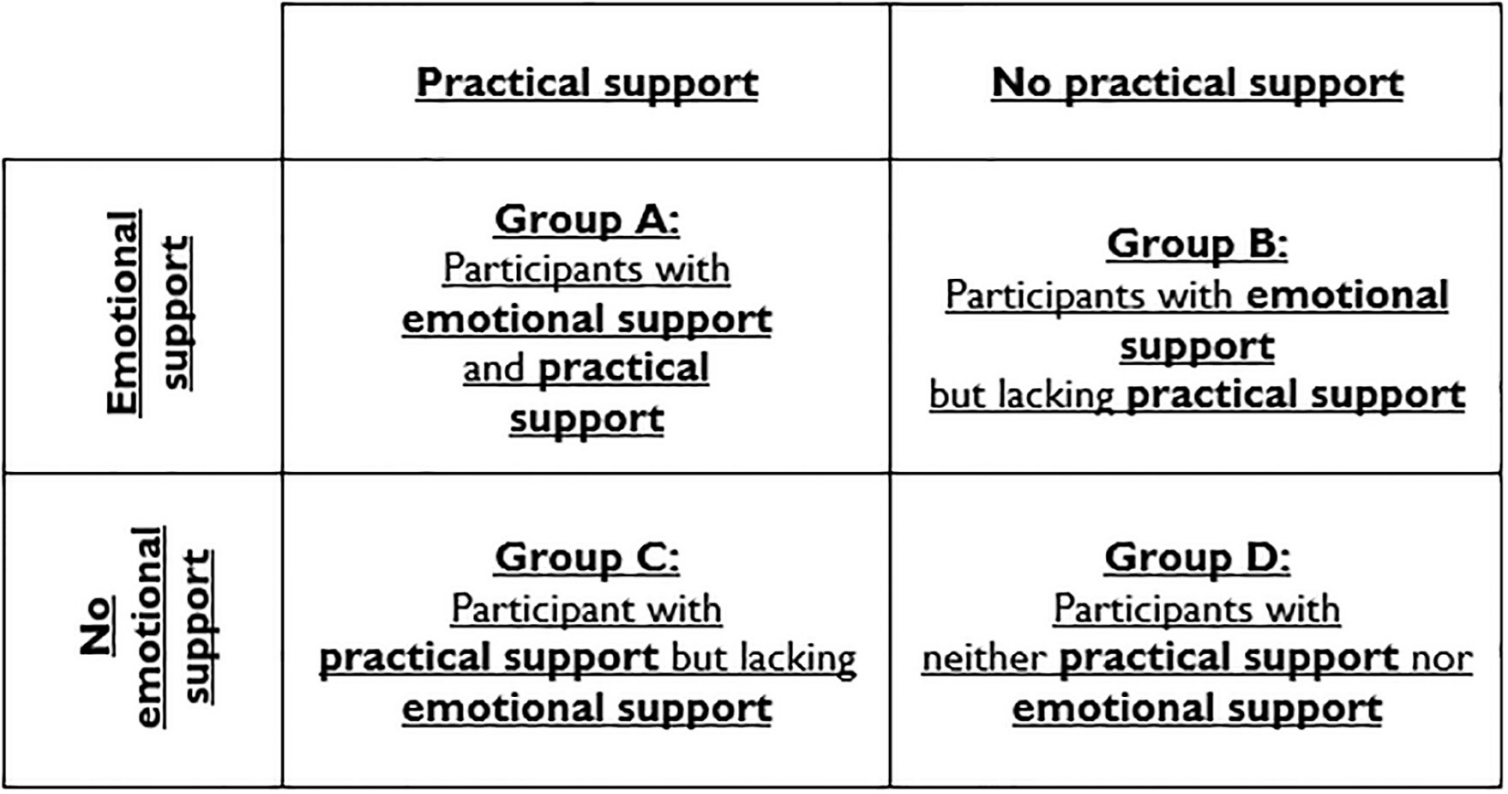
Stress support matrix.

Research has indicated that that practical support is an important element of the stress process as it aids in relieving distress [64,65]; this facet of support was for not evident in the data. Most participants had some social support in place but lacked the practical support that was needed to overcome the issues that they were facing. The overall deficiency of practical support within networks meant that participants needed to search for assistance from other sources. This led to a ‘support predicament’, in which participants had one form of support but not the other. This prevented them from being fully equipped to deal with incoming stressors. The Stress Support Matrix in Fig 1 illustrates the ‘support predicament’ and the different groups that are formed as a result of the differentiation between social and practical support.

The support group paradigm within this study can be viewed through the Stress Support Matrix. The participants in this study were mainly within Group B before they accessed CA and once practical support had been established, they then progressed into Group A. Some participants had been in Group D, however, they only moved into Group C as they still lacked full-time emotional support, in spite of their trusting relationship with the advisors.

#### Holistic model of welfare stress and coping

**Fig 2:**
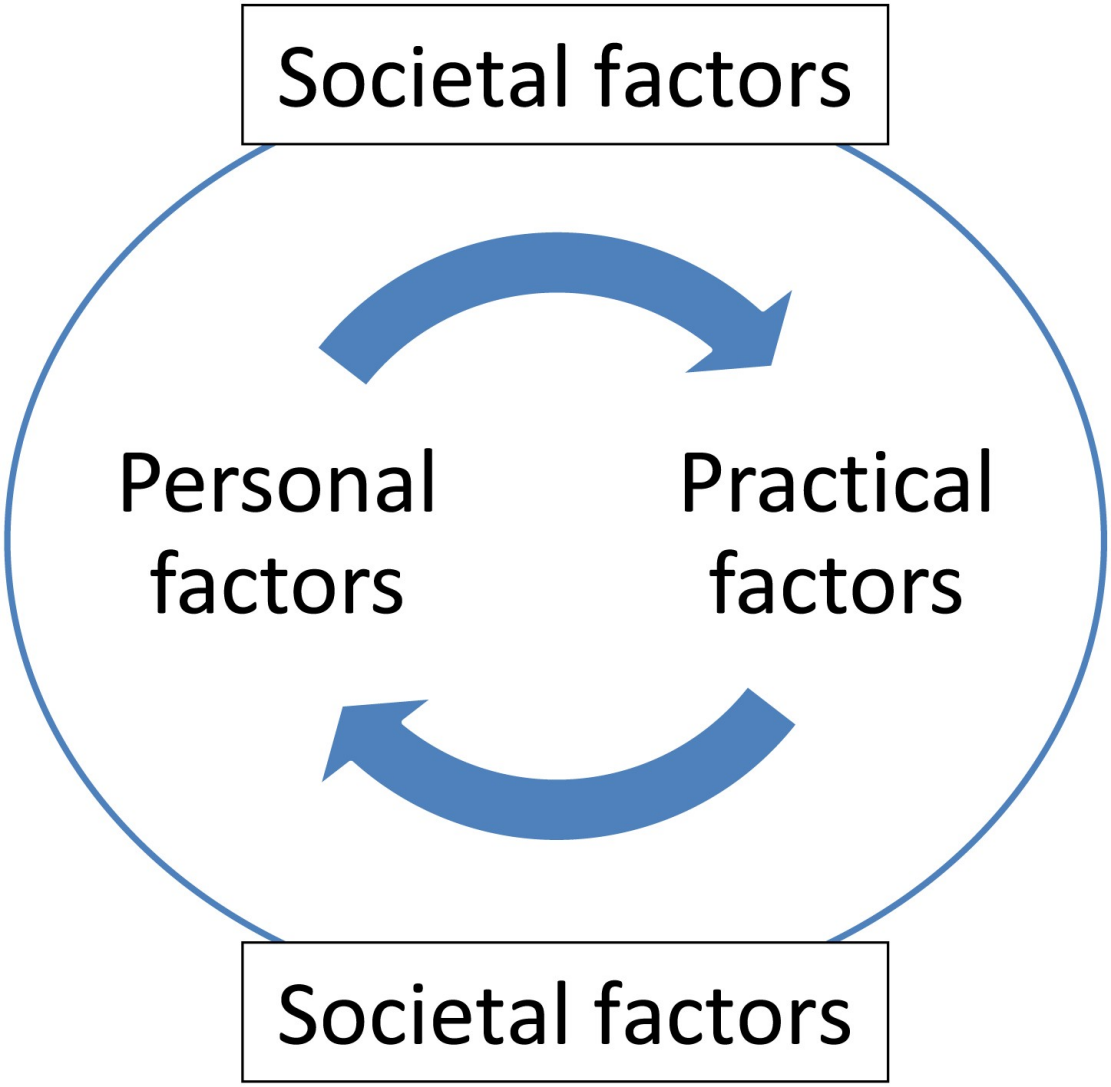
The holistic model of welfare stress and coping.

The findings can also be explained using on a multi-layered perspective focussing on personal, practical and societal levels (Fig 2).

The Personal is the most important level. This includes the individual’s past experiences of stress, their coping mechanisms and current stress schema. It also includes personal connections and emotional support. Stress is caused by the appraisal of the situation that participants find themselves in and whether they personally feel able to deal with it. At this level participants need to have an attitude which is conducive to seeking help in relieving stress. These include hardiness [50], locus of control [51], self-efficacy [52] and sense of coherence [53]. To reassure the individual of the personal aspect, services should prioritise and listen to their needs. CA did this by ensuring that their clients had a specific advisor as a point of referral, advisors listened carefully to the participants and participants felt their needs would be met.

The Practical is the second level. This includes the assistance that welfare advice seekers needed in working to resolve the stress caused by insufficient welfare support. Stress is felt by the lack of ability in being able to complete the necessary forms or understanding the process. CA ensured that their staff were well-versed in welfare legislation and had an expert level knowledge in the processes and procedures of the welfare system.

The Societal is the final level. This is the wider aspects of the welfare system; it includes the DWP, Jobcentre and society at large. It looks at the culture of welfare organisations, how they act towards welfare recipients and deal with their claims, and whether they are stigmatised. This level is the most remote from individual participants, as when they have personal and practical support, societal impacts are minimised. However it can still lead to stress as decisions rest with factors beyond the individual’s control. At this level, stigma can cause stress, with potential social isolation and exclusion. It can also preclude those that are entitled to welfare from applying [66,67], which results in non-uptake of benefits exacerbating insecurity and stress for those that need it.

### Limitations

The causes and experiences of stress are individual, intricate and multifaceted [17], and thus there is no consensual definition of stress. While a definition was chosen for this research, this inevitably restricts the researcher’s ability to engage in a thorough and dynamic inspection into stress theory and its experience.

This study was a secondary analysis, meaning that opportunities to explore stress in further detail with participants, and elaborate on particular points was limited. However, stress focused interviews might have led to data that was theory-driven and included leading questions which would have reinforced past research. The broad nature of the data allowed for an independent and objective analysis to build themes organically and it was true to what the participants had felt.

The study was not able to reach welfare claimants that had not accessed CA for practical support. It would have been worth having a group for comparison purposes, however, as they are a hard to reach target population, it would have been difficult to recruit.

### Implications

Participants had indicated that they feel welcome to use CA as resource in the future, while this is beneficial, there is a possibility that participants may become reliant on the service. In the long-term this could put pressure on the service and subsequently affect the value and level of assistance provided. While there has previously been points of high influx in the history of the organisation [30], the potential for the service to become overstretched and a subsequent decline in support remains.

Finally, the study highlights that action needs to be taken to reduce the stress experienced by welfare claimants through societal, as well as personal and practical factors, such as application and appeals processes and social stigma. Participants have clearly stated that their experiences of stress start from the beginning of their interaction with state organisations, until the end of the process.

## Conclusion

The study showed a thorough insight into the stress experiences of people who are claiming for welfare benefits. There were clear theoretical links between the participants’ experiences and substantive theories of stress. Welfare claimants experience stress in a variety of ways. Their idiosyncratic life experiences shape how their stress schemas work, however, they are all united in that once they received the suitable support through CA, they were able to overcome their stress. Furthermore, they felt confident in using CA in future.

## Supporting information

S1 File (PDF)

S2 File (PDF)

S3 File (DOCX)
