# Supplementary material for: ‘Everything takes too long and nobody is listening’: Developing theory to understand the impact of advice on stress and the ability to cope
Source: PLoS One. 2020 Apr 23;15(4):e0231014. doi: 10.1371/journal.pone.0231014 (PMC7179918; doi:10.1371/journal.pone.0231014)
Supplement: S1 File — (PDF) [file pone.0231014.s001.pdf]

Downloaded from http://bmjopen.bmj.com/ on February 5, 2016 - Published by group.bmj.com

BMJ Open

Exposing the impact of Citizens Advice Bureau services on health: a realist evaluation protocol

N Forster,[1,2] S M Dalkin,[1,2] M Lhussier,[1,2] P Hodgson,[1] S M Carr[1,2,3]

To cite: Forster N, Dalkin SM, Lhussier M, *et al*. Exposing the impact of Citizens Advice Bureau services on health: a realist evaluation protocol. *BMJ Open* 2016;**6**:e009887. doi:10.1136/bmjopen-2015-009887

▶ Prepublication history for this paper is available online. To view these files please visit the journal online (http://dx.doi.org/10.1136/bmjopen-2015-009887).

Received 3 September 2015
Revised 11 November 2015
Accepted 26 November 2015

CrossMark

[1]Department of Public Health and Wellbeing, Northumbria University, Newcastle Upon Tyne, UK
[2]Fuse (The Centre for Translational Research in Public Health), Newcastle University, Newcastle Upon Tyne, UK
[3]Federation University, Australia

Correspondence to
N Forster; natalie2.forster@northumbria.ac.uk

ABSTRACT

Introduction: Welfare advice services can be used to address health inequalities, for example, through Citizens Advice Bureau (CAB). Recent reviews highlight evidence for the impact of advice services in improving people's financial position and improving mental health and well-being, daily living and social relationships. There is also some evidence for the impact of advice services in increasing accessibility of health services, and reducing general practitioner appointments and prescriptions. However, direct evidence for the impact of advice services on lifestyle behaviour and physical health is currently much less well established. There is a need for greater empirical testing of theories around the specific mechanisms through which advice services and associated financial or non-financial benefits may generate health improvements.

Methods and analysis: A realist evaluation will be conducted, operationalised in 5 phases: building the explanatory framework; refining the explanatory framework; testing the explanatory framework through empirical data (mixed methods); development of a bespoke data recording template to capture longer term impact; and verification of findings with a range of CAB services. This research will therefore aim to build, refine and test an explanatory framework about how CAB services can be optimally implemented to achieve health improvement.

Ethics and dissemination: The study was approved by the ethics committee at Northumbria University, UK. Project-related ethical issues are described and quality control aspects of the study are considered. A stakeholder mapping exercise will inform the dissemination of results in order to ensure all relevant institutions and organisations are targeted.

Strengths and limitations of this study

- The use of realist evaluation enhances understanding of the underlying mechanisms through which advice services impact on health which are currently underexplored in existing literature.
- The study timescale inevitably restricts the empirical measurement of very distal health outcomes, which may be deemed a limitation of the study. However, the application of theory in realist evaluation enables attention to how proximal outcomes may lead to more distal outcomes further along the chain of causality.
- Working with practice partners to develop existing systems for recording health outcomes beyond the project timescale is a key strength of the study, ensuring impact of the research.

INTRODUCTION

There is established evidence linking poverty with inequalities in health and poorer life expectancy.[1] The impact of poverty on health is likely to have been exacerbated by recent reforms affecting disadvantaged groups disproportionately.[2] In targeting action on the wider determinants of health by promoting access to benefit entitlements and addressing housing and employment issues, for example, welfare advice services such as Citizens Advice Bureaux (CAB) services are one public health measure used to address health inequalities.[3 4] CAB deliver advice services from over 3300 community locations in England and Wales, run as a network of 338 individual charities, which together form the Citizens Advice service.[5] It is the responsibility of each individual CAB to secure its funding, which comes from a range of sources, including local government, non-governmental or charitable agencies, and clinical commissioning groups. Over 22 000 of the staff employed by Citizens Advice service are volunteers.[5] CAB provide 'independent, impartial, confidential and free advice to everyone on their rights and responsibilities',[6] including, but not limited to, advice on debt, benefits, employment, housing and discrimination. Gateshead CAB is one bureau of the Citizens Advice service, commissioned to offer advice to anyone who lives or works in Gateshead, which has a population of 202 000. Last year, Gateshead CAB provided advice to 13 235 clients (around 7%, of Gateshead's adult population) on over 43 835 issues.[7] The service

Downloaded from http://bmjopen.bmj.com/ on February 5, 2016 - Published by group.bmj.com

provides general advice through a walk-in service and telephone advice line, as well as delivering a number of projects which provide more intensive support for clients with complex needs and who are referred through the general service, or by health or social care practitioners. It is the latter, more intensive projects that form the focus of this evaluation.

Recent research highlights evidence for the impact of advice services in improving people's financial position and reducing poverty,[8–10] mental health and well-being, daily living and social relationships.[11 12] There is also some evidence for the impact of advice services in increasing accessibility of health services,[10] and reducing general practitioner (GP) appointments and prescriptions.[3 10] However, direct evidence for the impact of advice services on lifestyle behaviour and physical health is currently much less well established.[8 9] The current lack of evidence of impact on health is likely to be attributable to challenges in establishing such evidence, as opposed to an indication of lack of effect.[8]

CAB services take a holistic approach which attends to the multifaceted nature of vulnerability,[13] and the interconnectedness of social, economic and health difficulties.[14] As such, there is potential for CAB services to generate diverse and complex pathways of impact according to client circumstances, the number of issues for which support is sought and the categories of advice provided. Yet, 'advice services' have, for the most part, been treated homogenously within the literature, with little examination of the differential forms of service encompassed under this heading, how these may be tailored to the needs of different clients or groups, and lead to different outcomes. Existing attempts to determine whether or not advice services are effective have tended to focus on financial impacts, and might have masked potentially differential and longer term health improvement effects. Countering this trend, a logic model was recently developed, beginning to identify the plausible routes between advice interventions, direct outcomes of advice services (such as increasing disposable income, managing debt, and help with housing or employment), and longer term health improvement outcomes (such as reduced stress and anxiety or changes to health behaviour).[9] In order to build on this, there is a need for empirical testing of the specific mechanisms through which advice services and associated financial or non-financial benefits may lead to health improvements.[15]

Objectives

This project will:

1. Develop an explanatory framework of programme theories informing how, why, for whom and in what circumstances advice services impact on health.
2. Refine and test the explanatory framework using both existing theory and the generation and analysis of empirical data.
3. Identify and explain the contextual factors (eg, societal norms, client characteristics, adviser characteristics, delivery format) most likely to contribute to intervention effectiveness.
4. Map out the resources offered by CAB services likely to trigger a change in client reasoning.
5. Use findings on the health outcomes most likely to be achieved to inform existing CAB data recording systems with a view to capturing longer term impact on health.

METHODS AND ANALYSIS

Realist evaluation will be used to explore how, why, for whom and in what circumstances[16] CAB services are effective in improving health, using Gateshead CAB as an exemplar. Realist evaluation is a theory-driven approach which seeks to understand not only whether an intervention works, but also the detailed mechanisms leading to success or otherwise.[17] It acknowledges that interventions take place within complex social systems[16] and is therefore well suited to studying interventions, such as advice services, with complex and potentially multiple pathways from implementation to impact. The shorthand of context+mechanism=outcome (C+M=O) is used to express this, with mechanisms consisting of resources and reasoning.[16 18] Intervention resources (M) are introduced in a context (C), in a way that enhances a change in reasoning (M). This alters the behaviour of participants, which leads to outcomes (O).[18] As it has for other evaluations of holistic interventions,[19] the use of a realist approach will help to expose the multiple resources delivered under the umbrella of 'advice services', the ways that these may be employed in different contexts, and how these generate different outcomes.

The logic model established in by Allmark *et al*[9] shows links between immediate, intermediate and long-term outcomes. The use of a realist approach will add to this through tracing individuals' pathways from receipt of advice services through to intermediate and longer term outcomes, thereby exposing the causal mechanisms linking advice services to health-related outcomes. Key to a realist approach is the adjudication between mechanisms to decide which are significant in leading to an outcome, therefore enabling the impact of the multiple or potentially competing mechanisms identified earlier to be disentangled. Realist methods support the development and use of 'middle range theories',[20] which will help to generate transferable learning on the combination of contextual conditions and resources necessary to generate positive outcomes in advice services. Realist evaluation is suitable for use with complex interventions; advice services are complex interventions in that they are tailored to local contexts, are not delivered in isolation of other services or interventions, and as their outcomes in terms of health improvement are evident only after a long timescale.[9]

This research will therefore aim to build, refine and test an explanatory framework about how CAB services

Downloaded from http://bmjopen.bmj.com/ on February 5, 2016 - Published by group.bmj.com

can be optimally implemented to achieve health improvement.

Operationalisation of the project: five phases

The operationalisation of realist methods has been described as challenging by several researchers.[18] Here we outline how we will operationalise this method, in five phases.

Phase 1: building the explanatory framework

This phase will utilise the experience of key stakeholders to develop programme theories of how and why CAB services generate health outcomes. Programme theories will be informed by interviews with CAB staff, who will be selected purposively according to their involvement with specific projects to be evaluated. Interviews will be undertaken in parallel and will focus on making explicit for each separate project (1) the contextual influences on CAB projects; (2) the resources provided by CAB (eg, the types and topics of advice delivered); (3) the changes in clients' reasoning that these resources trigger (eg, decisions about how to use increased disposable income, psychosocial responses); and (4) how these components work together to generate health improvement. The same advice service resource may impact differently on the reasoning of clients depending on the particular project target group, for instance whether or not they are living with a long-term condition and/or have difficulty leaving their home. Interviews will be audio recorded and transcribed, and data coded according to contextual influences, programme resources, participant reasoning, outcomes and where possible links between these different components. Programme and middle range theories will then be developed by the research team, during collaborative meetings based on these findings. Programme theories will be expressed as context–mechanism–outcome configurations (CMOc). For example, one of the specific programme theories developed states: *In a society where there is a culture of shaming, the unhealthy (context) increased finances (resource) result in increased self-efficacy to be healthy (buy healthy items/do healthy activities; reasoning). This results in using the additional finances secured as a result of accessing CAB to buy/access healthy items and engage in healthy activities (outcome).* One of the overarching middle range theories states: *In a context of neoliberalism, advice (resource) leads to increased knowledge about rights and a feeling of support in CAB clients, enabling them to challenge people in authority (reasoning) leading to increased confidence to take action (outcome 1) and reduction in stress (outcome 2).* The process will be iterative, moving back and forth between the data generated and emerging programme, middle range and formal theories in order to ensure that they are substantiated. A second round of interviews and/or focus groups with CAB project staff will be undertaken in order to discuss, test and refine the initial programme theories developed. It is recognised that no single evaluation can refine and test all possible programme theories about how advice services work, for whom, in what circumstances and why. A key part of the development and refining of programme theories undertaken in conjunction with CAB staff in phases 1 and 2 will be to clarify the focus of the evaluation and specify the key theories that this evaluation will seek to test (including decisions about how far along the implementation chain evidence on impact will be sought). The result of this phase will be a comprehensive, but not exhaustive list of programme theories which will together form an overarching model (the explanatory framework) of how, when or in which circumstances advice services lead to different outcomes that will be refined and empirically tested throughout subsequent stages. This set of programme theories will also include 'middle range theories' which describe how advice services in general can lead to health improvement.

Phase 2: refining the explanatory framework

Broader literature will be used to refine and substantiate the realist programme and middle range theories, thereby clarifying the causal pathways between advice service components and health outcomes, depending on particular contexts.[21] For example, theories may be drawn on which suggest that increased income may improve health through material, psychosocial or behavioural mechanisms.[22] Literature will be located through searches of social science and health databases, as well as through checking the reference lists and citations of relevant studies. Consistent with realist approaches, searches of the literature will be undertaken purposively and iteratively, with publications selected according to their ability to refine programme theories. Search terms to be used will be generated through discussion among the project team. This process will involve identifying the key concepts and processes suggested to have explanatory power within the programme theories developed in phase 1, before translating these into specific search terms and strategies.

Phase 3: testing the explanatory framework through empirical data

A mixed-methods approach will be utilised to empirically test the explanatory framework. Quantitative analysis will be undertaken of both existing CAB data on client demographics and outcomes, as well data collected through the project on health outcomes, using interviews, the Perceived Stress Scale,[23] the 14-item Warwick Edinburgh Mental Wellbeing Scale (WEMWBS)[24] and questions on changes to lifestyle. Qualitative data will be generated through interviews with CAB clients to explore changes in health outcomes and the reasoning mechanisms undertaken. Qualitative interviews will also explore if and how other sources of advice have been drawn on and the reasoning processes through which this advice was reconciled with that received through CAB. This is in order to better understand the distinct contribution that CAB advice may make. Findings from

Downloaded from http://bmjopen.bmj.com/ on February 5, 2016 - Published by group.bmj.com

previous interviews and from quantitative data on the health outcomes generated in different circumstances, where collected prior to interviews, will also be used to guide further qualitative interview schedules. The evaluation will sample from three projects offered by Gateshead CAB: one targeting young people aged between 16 and 25; one targeting people experiencing severe and enduring mental health difficulties; and one for patients referred by GP practice staff who have difficulty leaving their home due to, for example, disability or caring responsibilities. For the quantitative strand of the evaluation, all new clients referred to these services will be asked to complete the selected health measures, in addition to the data routinely collected by CAB. Based on average monthly client referral rates to each of the projects included in the evaluation, it is estimated that health outcome measure data (the Perceived Stress Scale and the WEMWBS) will be collected for around 480 CAB clients and linked to data routinely collected by CAB, including client demographics, number and type of advice issues that clients approached with, number of contacts with CAB, financial benefits (eg, increased disposable income) and non-financial benefits (eg, food parcels) received. We also have access to retrospective WEMWBS data collected by CAB for one of the projects being evaluated.

Where possible, as it is understood that this population is transient, purposive sampling will be used to select clients to be invited to participate in two qualitative interviews. Sampling criteria will be developed based on the contextual influences, advice service resources and outcomes that emerge as important throughout phases 1 and 2. It is estimated that we will undertake interviews with up to 10 clients of each project being investigated. However, flexibility will be exercised over the precise number of interviews to be undertaken, guided by the number of cases necessary to test the different permutations of contexts mechanisms and outcomes in the programme theories under study.[25] We acknowledge that engaging CAB users in the research process might be a challenge, and will be working closely with CAB staff to elicit the best ways of doing so. The outcome of phase 3 of the research will be a refined explanatory framework which is both theoretically grounded and empirically substantiated.

Quantitative data analysis will be used to test programme theories about the outcomes that advice services are expected to generate, and how these outcomes may differ according to particular contexts or for particular clients. There are a number of challenges to generating data on the outcomes of advice services owing to variation in client patterns of accessing advice services, differences in the timescales after which clients receive any benefits, and difficulties in retention to follow-up due to population transience. The precise analytic method for the study is therefore contingent on the strength of the data that can be collected and the final sample sizes. A descriptive analysis will be undertaken of quantitative data on changes between baseline and follow-up. In the case that there is sufficient quality of data to allow it, regression analysis will be undertaken in order to estimate relationships between independent variables (such as the particular client group, or benefit received) and health outcomes, and to distinguish between the differential effects of these independent variables on health outcomes when the others are held constant. Where there is insufficient data to undertake a regression analysis, exploratory analysis using t tests will be undertaken to consider how mechanisms produce outcomes in an accompanying context, as per the methodology.

Qualitative analysis focuses on testing theories on the underpinning mechanisms (in the form of the reasoning of clients) that generate particular outcomes. Following previous approaches to analysis of qualitative data for the purposes of realist evaluation,[26] examples of 'dyads, triads or more complex strings' of CMOc in participant's narratives will be identified and coded. These configurations will then be scrutinised in order to establish patterns in the mechanisms or groupings of mechanisms together with certain contexts that lead to the same outcomes.

Phase 4: development of a bespoke data recording template to capture longer term impact

Evidence from phase 3 on the most likely health improvement outcomes resulting from CAB services will be used to develop a bespoke package of data collection measures that can be used by CAB in order to facilitate in capturing health impacts beyond the evaluation timescale. Researchers will work with CAB staff, as well as the Citizen's Advice Service Design, Development and Innovation Team in order to ensure that the template is suitable for embedding in routine practice.

Phase 5: verification of findings with a range of CAB services

The Citizen's Advice Service Design, Development and Innovation Team are about to embark on a project to support Bureau Design Champions to develop new approaches to delivering health-related services, including support with prototyping in-depth research and impact measurement. As such, there is potential for wide scale interest and applicability of the explanatory framework and data recording template. This stage will involve three verification events with wider CAB stakeholders in order to explore the transferability of findings to different service formats.

As approaches to generating evidence in this area are in their infancy, and as the distal nature of health outcomes means they are unlikely to be captured within the study timescale, this project does not seek to produce definitive answers about the effectiveness of advice services on physical health. Rather, it presents an innovative approach to develop greater understanding of how the complexity of advice service outcomes can be captured

Downloaded from http://bmjopen.bmj.com/ on February 5, 2016 - Published by group.bmj.com

and evaluated. The use of realist evaluation, with an emphasis on using and developing theory, enables explanations to be built of how immediate and intermediate outcomes from CAB (in this case reduced stress and increased well-being), may in turn lead to health outcomes occurring later in the causal chain. In addition, eliciting the underpinning mechanisms that link immediate, intermediate and longer term outcomes can assist in better delineating the role of advice services in improving health amid other extraneous factors. Finally, the incorporation of a service development aspect to inform existing data recording systems facilitates ongoing measurement of likely health impacts beyond the study timescale.

ETHICS AND DISSEMINATION

Ethics

Project data will be stored on a secure University drive for 3 years postproject completion for potential audit purposes.

It is acknowledged that those participating in interviews may be classed as vulnerable as they may still be experiencing financial or related health issues. In order to ensure appropriate questions are posed to these participants, interview schedules will be sent to CAB staff for comment and refinement. Invitations to CAB clients will be sent via CAB staff and interviews will take place at Gateshead CAB as it is familiar to both the participant and the researchers. There is some risk that participants may become upset when participating in data collection. Participants will be informed in advance of the broad areas to be discussed during interviews in order that they can make an informed decision about whether or not they want to take part. Participants will be assured that they do not have to answer any questions that they do not want to, and should a person become upset or distressed, they will be given the option of ceasing participation. Researchers will also equip themselves with information about local support services, in addition to CAB which can be provided to participants where appropriate. Participants will be reimbursed for their travel to and from Gateshead CAB. All interview recordings and transcripts will be given pseudonyms, and the list of respondents and their pseudonyms will be kept separately from the data. At all stages of the study, data will be kept strictly confidential and findings will be reported anonymously.

All participants in the study (CAB staff and clients) will provide informed consent after reading a detailed yet easy to understand information sheet. In cases where CAB clients are illiterate, participant information sheets and consent forms will be described in full to participants verbally. Audio copies of the information will also be housed at Gateshead CAB. Before the interview, time will be dedicated to oral explanation of the content and to answer any questions the interviewee might have. Participants will have the right to decline participation after reading the information sheet and will be reminded of their right to withdraw after the interview, stressing that it will not affect the services they receive in future from CAB.

Quality control

In order to increase the validity of our initial programme theories, several steps will be taken. First, the team will map the implementation process of CAB services. Second, programme theories will be developed in regular team meetings in order to allow a process of retroduction and questioning, which will facilitate the development of robust programme theories. Third, in order to be transparent in the generation of programme theories, as encouraged by Wong *et al*,[20] an audit trail of the debate, refinement and decision-making undertaken by the team will be captured for each theory using NVivo. Fourth, the final set of initial programme theories will be sent to CAB staff for refinement. Qualitative interview analysis will be cross checked by three researchers (SMD, NF, PH). A similar team meeting process will be used for programme theory refinement in order to ensure robust testing and refinement of the programme theories.

Difficulties in operationalising realist methods have been reported.[18] This project precedes the publication of the Realist And Meta-narrative Evidence Syntheses: Evolving Standards (RAMESES) publication standards for realist evaluation (RAMESES II). In order to ensure quality, the team will therefore regularly engage with realist literature, the RAMESES II project (as it develops), realist experts in the field and realist training events and conferences. The team also have considerable experience in realist evaluation.[27–30]

As a team, we recognise that it will be difficult to find specific health outcomes when theorising about how CAB works, for whom, in which circumstances. Often health outcomes will be more distal, occur over a longer time period or be the result of CAB input in collaboration with other services (eg, primary care). Interviews will include a general question about whether clients have received advice from other sources. Thus, when collecting data, CAB clients will be asked to answer questions about changes to health in relation to CAB specifically, as opposed to other services they may have accessed, in order to understand the distinct contribution the service makes (if any) to their health. Furthermore, a focus on stress, anxiety and depression will be taken as these conditions are likely to be impacted by CAB intervention with more immediate effect. There is a plethora of research to link stress, anxiety and depression with other aspects of physical health.[31–35] Using this mental health, lens will allow us to theorise on the distal physical health impacts CAB may have, which cannot be documented through empirical data collection.

Downloaded from http://bmjopen.bmj.com/ on February 5, 2016 - Published by group.bmj.com

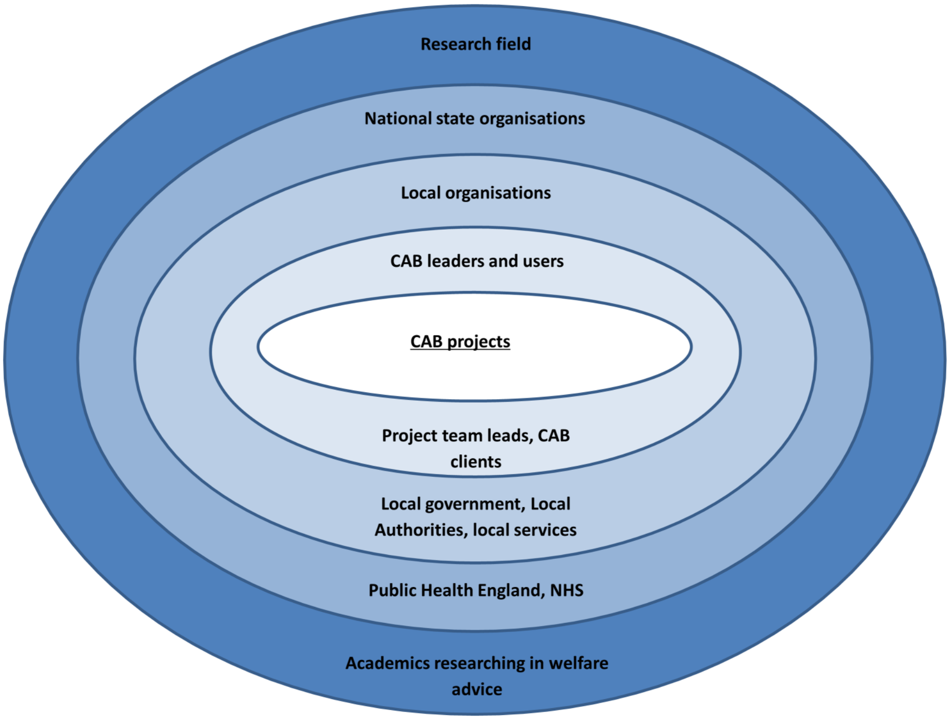

Figure 1 Stakeholder view of the corporation, adapted from Post *et al*.[36] CAB, Citizens Advice Bureau; NHS, National Health Service.

Dissemination

Dissemination will be guided by figure 1. This figure encompasses the relevant stakeholders of corporations (used in the original to refer to firms or business corporations but applied here to CAB) and thus will ensure dissemination is targeted at all levels. Academics researching in welfare will be targeted through publications and conference presentations. National state organisations such as Public Health England and the National Health Service (NHS) will be targeted through submission of oral presentations at Public Health England Annual Conference and the UK Clinical Research Collaboration (UKCRC) Public Health Research Centres of Excellence Conference in 2016. Local government, local authorities and local services will be provided with information about the project findings via a dissemination event postproject completion, which all stakeholders will also be invited to. Information and findings will also be circulated to public health practitioners via the opportunities available through Fuse (the Centre for Translational Research in Public Health) and distributed in the monthly e-newsletter from the Association of Directors of Public Health. CAB staff will be involved with the project throughout, as is recommended in realist evaluation,[16] and therefore dissemination will be ongoing to this group. They will also be invited to contribute to publications. All levels of the stakeholder view of the corporation will be represented in the project stakeholder group.

Acknowledgements The authors wish to thank the reviewers for their insightful comments on this paper, which have helped to improve the manuscript.

Contributors SMC, NF and ML conceived of the study design. SMD and NF led on the protocol with editing comments from ML, PH and SMC.

Funding This work was supported by the National Institute for Health (NIHR) School for Public Health Research (SPHR), through the Public Health Practice Evaluation Scheme (PHPES).

Competing interests None declared.

Ethics approval Northumbria University Research Ethics Committee (HLS-PHW141524).

Provenance and peer review Not commissioned; externally peer reviewed.

Open Access This is an Open Access article distributed in accordance with the terms of the Creative Commons Attribution (CC BY 4.0) license, which permits others to distribute, remix, adapt and build upon this work, for commercial use, provided the original work is properly cited. See: http://creativecommons.org/licenses/by/4.0/

REFERENCES

1. Office for National Statistics. *Healthy life expectancy at birth for upper tier local authorities: England, 2010–12*. London: Office for National Statistics, 2014.
2. Inquiry Panel on Health Equity for the North of England. *Due north: the report of the inquiry on health equity for the north*. Great Britain: University of Liverpool and Centre for Local Economic Strategies, 2014.
3. Palmer S, Dalzell-Brown A, Mather K, *et al*. *Evaluation of the impact on GP surgeries of the Citizens Advice Bureau Health Outreach Service*. Sefton: NHS Sefton, 2010.
4. Hirst J, Minter S. *Citizens Advice Bureaux in General Practice Report 2013/14*. Derbyshire: Derbyshire County Council and Citizens Advice Bureau. 2014.
5. Citizens Advice. Introduction to the Citizens Advice Service (cited 21 October 2015). https://www.citizensadvice.org.uk/about-us/how-citizens-advice-works/who-we-are-and-what-we-do/introduction-to-the-citizens-advice-service/
6. Citizens Advice. About Citizens Advice (cited 21 October 2015). https://www.citizensadvice.org.uk/about-us/
7. Gateshead Advice Centre. *Challenges, choices, change: annual review 2013–2014*. Gateshead: Gateshead Advice Centre, 2014.
8. Adams J, White M, Moffatt S, *et al*. A systematic review of the health, social and financial impacts of welfare rights advice delivered in healthcare settings. *BMC Public Health* 2006;6:81.

Downloaded from http://bmjopen.bmj.com/ on February 5, 2016 - Published by group.bmj.com

9. Allmark P, Baxter S, Goyder E, *et al*. Assessing the health benefits of advice services: using research evidence and logic model methods to explore complex pathways. *Health Soc Care Community* 2013;21:59–68.
10. Citizens Advice Bureau. *An overview of possible links between advice and health*. London: Citizens Advice Bureau, 2012.
11. Burrows J, Baxter S, Baird J, *et al*. Citizens advice in primary care: a qualitative study of the views and experiences of service users and staff. *Public Health* 2011;125:704–10.
12. Citizens Advice Bureau. *What did CAB do for you?* London: CAB, 2014.
13. Aspinall P. *Hidden needs. Identifying key vulnerable groups in data collections: vulnerable migrants, gypsies and travellers, homeless people, and sex workers*. Kent, 2014.
14. Leeds City Council FIT. *Improving public health through income maximisation. Commissioning advice services best practice guide*. Leeds: Leeds City Council, 2011.
15. Rowlinson K. *Does income inequality cause health and social problems?* York, 2011.
16. Pawson R, Tilley N. *Realistic evaluation*. London: Sage, 1997.
17. Pawson R, Manzano-Santaella A. A realist diagnostic workshop. *Evaluation* 2012;18:176–91.
18. Dalkin S, Greenhalgh G, Jones D, *et al*. What's in a mechanism? Development of a key concept in realist evaluation. *Implement Sci* 2015;10:49.
19. Hardwick R. Integrated services for women through a One Stop Shop: a realist review. *J Integr Care* 2013;21:263–75.
20. Wong G, Greenhalgh T, Westhorp G, *et al*. RAMESES publication standards: realist syntheses. *BMC Med* 2013;11:21.
21. Davidoff F, Dixon-Woods M, Leviton L, *et al*. Demystifying theory and its use in improvement. *BMJ Qual Saf* 2015;24:228–38.
22. Benzeval M, Bond L, Campbell M, *et al*. *How does money influence health?* York: Joseph Rowntree Foundation, 2014.
23. Cohen S, Kamark T, Mermelstein R. A global measure of perceived stress. *J Health Soc Behav* 1983;24:385–96.
24. Stewart-Brown S, Tennant A, Tennant R, *et al*. Internal construct validity of the Warwick-Edinburgh Mental Well-being Scale (WEMWBS): a Rasch analysis using data from the Scottish Health Education Population Survey. *Health Qual Life Outcomes* 2009;7:15.
25. Emmel N. *Sampling and choosing cases in qualitative research: a realist approach*. London: Sage, 2013.
26. Jackson SF, Kolla G. A new realistic evaluation analysis method: linked coding of context, mechanism, and outcome relationships. *Am J Eval* 2012;33:339–49.
27. Carr S, Lhussier M, Forster N, *et al*. Outreach programmes for health improvement of Traveller Communities: a synthesis of evidence. *Public Health Res* 2014;2:1–168.
28. Lhussier M, Forster N, Carr SM. A realist synthesis of the evidence on outreach programmes for health improvement of Traveller Communities. *J Public Health* 2015:pii: fdv093.
29. Greenhalgh J, Pawson R, Wright J, *et al*. Functionality and feedback: a protocol for a realist synthesis of the collation, interpretation and utilisation of PROMs data to improve patient care. *BMJ Open* 2014;4:e005601.
30. Dalkin SM, Jones D, Lhussier M, *et al*. Understanding integrated care pathways in palliative care using realist evaluation: a mixed-methods study protocol. *BMJ Open* 2012;2:e001533.
31. Wilson MD, Conroy LM, Dorevitch S. Occupational stress and subclinical atherosclerosis: a systematic review. *Int J Occup Environ Health* 2014;20:271–80.
32. Fond G, Loundou A, Hamdani N, *et al*. Anxiety and depression comorbidities in irritable bowel syndrome (IBS): a systematic review and meta-analysis. *Eur Arch Psychiatry Clin Neurosci* 2014;264:651–60.
33. Hintsa T, Shipley MJ, Gimeno D, *et al*. Do pre-employment influences explain the association between psychosocial factors at work and coronary heart disease? The Whitehall II study. *Occup Environ Med* 2010;67:330–4.
34. Backé EM, Seidler A, Latza U, *et al*. The role of psychosocial stress at work for the development of cardiovascular diseases: a systematic review. *Int Arch Occup Environ Health* 2012;85:67–79.
35. Langan SM, Williams HC. What causes worsening of eczema? A systematic review. *Br J Dermatol* 2006;155:504–14.
36. Post JE, Preston LE, Sachs S. Managing the extended enterprise: the new stakeholder view. *California Manag Rev* 2002;45:6–28.

Downloaded from http://bmjopen.bmj.com/ on February 5, 2016 - Published by group.bmj.com

BMJ Open

Exposing the impact of Citizens Advice Bureau services on health: a realist evaluation protocol

N Forster, S M Dalkin, M Lhussier, P Hodgson and S M Carr

BMJ Open 2016 6:
doi: 10.1136/bmjopen-2015-009887

Updated information and services can be found at:
http://bmjopen.bmj.com/content/6/1/e009887

These include:

References This article cites 20 articles, 5 of which you can access for free at:
http://bmjopen.bmj.com/content/6/1/e009887#BIBL

Open Access This is an Open Access article distributed in accordance with the terms of the Creative Commons Attribution (CC BY 4.0) license, which permits others to distribute, remix, adapt and build upon this work, for commercial use, provided the original work is properly cited. See: http://creativecommons.org/licenses/by/4.0/

Email alerting service Receive free email alerts when new articles cite this article. Sign up in the box at the top right corner of the online article.

Topic Collections Articles on similar topics can be found in the following collections

Health services research (854)
Public health (1377)
Research methods (369)

Notes

To request permissions go to:
http://group.bmj.com/group/rights-licensing/permissions

To order reprints go to:
http://journals.bmj.com/cgi/reprintform

To subscribe to BMJ go to:
http://group.bmj.com/subscribe/
