# Supplementary material for: ‘Everything takes too long and nobody is listening’: Developing theory to understand the impact of advice on stress and the ability to cope
Source: PLoS One. 2020 Apr 23;15(4):e0231014. doi: 10.1371/journal.pone.0231014 (PMC7179918; doi:10.1371/journal.pone.0231014)
Supplement: S2 File — (PDF) [file pone.0231014.s002.pdf]

Received: 23 April 2018 | Revised: 5 November 2018 | Accepted: 8 November 2018

DOI: 10.1111/hsc.12695

ORIGINAL ARTICLE

WILEY Health and Social Care in the community

Exposing the impact of intensive advice services on health: A realist evaluation

Sonia Michelle Dalkin[1,2] | Natalie Forster[1,2] | Philip Hodgson[3] | Monique Lhussier[1,2] | Pete Philipson[4] | Susan Mary Carr[1,2,5]

[1]Department of Social Work, Education and Community Wellbeing, Northumbria University, Benton, Newcastle Upon Tyne, UK

[2]Fuse (The Centre for Translational Research in Public Health), Institute of Health & Society, Newcastle University, Newcastle Upon Tyne, UK

[3]Department of Nursing, Midwifery and Health, Northumbria University, Benton, Newcastle Upon Tyne, UK

[4]Department of Mathematics, Physics and Electrical Engineering, Northumbria University, Newcastle upon Tyne, UK

[5]Department of Education and Training, Federation University Australia, Mount Helen, Victoria, Australia

Correspondence
Sonia Michelle Dalkin, Department of Social Work, Education and Community Wellbeing, Northumbria University, Benton, Newcastle Upon Tyne, UK.
Email: s.dalkin@northumbria.ac.uk

Funding information
This work was supported by the NIHR's School for Public Health Research (SPHR) [project reference: SPHR-FUS-PES-CAB].

Abstract

Attention has turned to welfare advice as a potential health and social care intervention. However, establishing direct evidence of health impact has proven difficult. This is compounded by the need to understand both the facilitative contexts and mechanisms through which this impact occurs. This study investigated if, how and in which circumstances an intensive advice service had an impact on stress and well-being (as precursors to health impacts), for clients attending a branch of Citizens Advice, located in the North East of England. A mixed methods realist evaluation of three intensive advice services offered by Citizens Advice (CA) was operationalised in five phases: (a) Building programme theories, (b) refining programme theories, (c) Development of a data recording tool, (d) Testing programme theories with empirical data, (e) Impact interviews. This paper focuses on phase 4. The Warwick Edinburgh Mental Wellbeing Scale (WEMWBS) and Perceived Stress Scale (PSS) were completed by 191 clients, with a 91% follow-up rate (data collected: February 2016 to March 2017). Twenty-two CA clients participated in interviews (data collected: October 2015 to November 2016). The PSS indicated a significant decrease in stress from initial consultation to approximately 4–6 weeks post advice from 31.4 to 10.3 ($p < 0.001$) and the WEMWBS indicated a significant increase in client well-being from a mean of 26.9 to 46.5 ($p < 0.001$). Nine refined programme theories are presented which combine the qualitative and quantitative analysis; they are underpinned by three abstract theories: Capabilities model, The Decision to Trust Model, and Third Space. An explanatory framework is presented covering the micro, meso, and macro levels of CA. Use of a stress and well-being lens has allowed insight into the precursors of health in those receiving intensive advice. Using these measures whilst explaining contextual and mechanistic properties, begins to build a complex and real picture of how advice services impact on health.

KEYWORDS
advice services, evaluation research, realist, stress, trust, well-being

 © 2018 John Wiley & Sons Ltd

1 | BACKGROUND

Following the formation of the coalition government in 2010 in the United Kingdom, a period of particularly significant change to welfare provision ensued (Moffatt et al., 2016). The Welfare Reform Act in particular, set out a number of austerity measures to reduce public spending on welfare support (Welfare Reform Act, 2012). The reforms were place-blind, ignoring geographical variation in claimant trends, housing provision, and labour markets (Edwards, Jarvis, Jarvis, Shaw, & Irving, 2013), and disproportionately affected the most disadvantaged areas of the United Kingdom, such as the North of England (Whitehead, 2014).

The impact of poverty on health has long been recognised (Acheson, 1988; Black, Morris, Smith, & Townsend, 1980 ; Marmot, 2010). Those living in the most deprived areas of England and Wales have lower life expectancy and live in good health for fewer years than those from more affluent areas (Office for National Statistics, 2014). Research also points to the relationship between poverty and mental health and well-being (Mental Health Foundation, 2016), with a reciprocal relationship between financial strain and mental health (Holkar & Mackenzie, 2016). The North of England has consistently poorer mortality rates than the rest of England; this gap has widened over four decades and under five governments (Hacking, Muller, Muller, & Buchan, 2011). In addition, the North East region of England has been shown to have the greatest prevalence of diagnosed mental health issues (Bridges, 2014) and the highest suicide rate (Office for National Statistics, 2015).

Given the recognised relationship between poverty, income, and health, public health attention has turned to welfare advice as a potential health intervention in its own right (Abbott, 2002; Woodhead, Khondoker, Khondoker, Lomas, & Raine, 2017). Welfare advice refers to support with many issues including benefits, housing, employment, debt, and money. However, establishing direct evidence on the impact of welfare advice on health has proven difficult using traditional forms of evaluation, for a number of reasons (Adams, White, White, Moffatt, Howel, & Mackintosh, 2006; Allmark, Baxter, Baxter, Goyder, Guillaume, & Crofton-Martin, 2013). The relationship between poverty and health is complex, working through a combination of material, psychosocial, and behavioural mechanisms (Abbott, 2002; Benzeval et al., 2014), for example, through a reduction in stress. In addition, the spectrum of individuals' experiences of poverty means that it can be difficult to develop conclusive evidence of how reducing poverty affects health (Abbott, 2002). That advice services themselves are examples of complex interventions, highly tailored to individual needs, further adds to this challenge. As a result and as identified in a previous article in this journal, the rationale for implementing welfare advice as a health intervention is often left implicit (Abbott, 2002).

More recently, research has sought to chart more precisely the routes through which welfare advice impacts on health (Allmark et al., 2013). Some evidence exists to substantiate these pathways. Existing research has demonstrated that advice services can lead to improvements in mental health and well-being for recipients (Abbott, Hobby, Hobby, & Cotter, 2005; Burrows, Baxter, Baxter, Baird, Hirst, & Goyder, 2011; Citizens Advice Bureau, 2012; Hirst & Minter, 2014; Moffatt & Scambler, 2008). However, further research is required to test these pathways empirically, and to understand the underpinning mechanisms through which advice services generate these health outcomes. A better understanding of how welfare advice operates is important in ascertaining the distinct role and value of advice services in improving people's health and reducing stress (both psychological and physiological (Gianaros & Wager, 2015)).

This paper reports findings from an evaluation of three projects situated within one Citizens Advice (CA) Service in the North East of England, United Kingdom. These projects, unlike the standard 20 min CA appointments, provided intensive support over a period of time (from 2 months to 2 years) to clients experiencing multiple and complex issues. These included (a) a project for people with severe and enduring mental health issues; (b) a project for people referred through their GP; and (c) a project for young people aged 16–25. All the projects, in effect, acted as the same intervention which was to three different client groups; the projects were separated due to different funding streams. As a result, the service provided constituted a complex intervention for those with complicated welfare issues. While the assistance offered by projects exceeds the provision of advice alone, with CA staff often adopting an advocacy role, as is common in this field, the term advice is used throughout the paper to encompass the range of support given. The mechanisms associated with, and the differential impact of specific forms of support is delineated in the reporting of results.

What is known about this topic

- Existing research has demonstrated that advice services can lead to improvements in mental health and well-being for recipients.
- The impact of poverty on health has long been recognised.
- Public health attention has turned to welfare advice as a potential health intervention in its own right.

What this paper adds

- Insights for practice and research on how the health outcomes of intensive advice services can best be captured.
- Understanding of the essential facilitative contexts and mechanisms within welfare advice leading to positive outcomes for clients.
- A theoretically driven explanation of advice services' impact on health is offered, at the mirco, meso, and macro level.

TABLE 1 Descriptions of mechanism, context and outcomes (Pawson & Tilley, 1997)

Mechanism	Mechanisms describe what it is about programmes and interventions that bring about any effects. It is not programmes that work, but the resources they offer to enable their subjects to make them work. This process of how subjects interpret and act upon the intervention stratagem is known as the mechanism.
Context	Mechanisms will only be active in particular circumstances, that is, in different contexts. Context describes those features of the conditions in which programmes are introduced that are relevant to the operation of mechanisms. Context must not be confused with locality; it can include cultural norms, economic conditions, existing public policy, for example.
Outcomes	Also known as outcome patterns. Outcome-patterns comprise the intended and unintended consequences of programmes, resulting from the activation of different mechanisms in different contexts. They can be proximal, intermediate, or distal.

The aim of this study was to identify if, how and in which circumstances an intensive advice service had an impact on stress and well-being (as precursors to health impacts).

2 | METHODS

Realist evaluations are used to further understand the impact of complex interventions; they go beyond questions of effectiveness, to highlight the contexts and mechanisms (including resources and reasoning) through which interventions function (Dalkin, Jones, Jones, Lhussier, & Cunningham, 2012; Pawson & Tilley, 1997). Context, mechanism, and outcome configurations (CMOC) are used as a heuristic by the researcher to develop realist informed programme theories about how the intervention is supposed to function (Greenhalgh et al., 2017); further details on each concept are given in Table 1. These theories are then tested using empirical data (Dalkin, Greenhalgh, Greenhalgh, Jones, Cunningham, & Lhussier, 2015). Realist evaluations are method neutral, often drawing on local effectiveness data to identify outcomes (Dalkin, Lhussier, Lhussier, Philips, Jones, & Cunningham, 2016; Dalkin, Lhussier, Lhussier, Williams, Burton, & Rycroft-Malone, 2018) and on qualitative insights for theory generation and refinement. In this instance, participants' well-being and stress were measured using two outcome measures; the Warwick Edinburgh Mental Wellbeing Scale (WEMWBS) (Tennant et al., 2007) and the Perceived Stress Scale (PSS) (Cohen, Kamarck, & Mermelstein, 1983) before and after advice (n = 191; 91% return of the post advice data collection), alongside semistructured, face to face qualitative realist interviews (Manzano, 2016) with staff (n = 11) and clients (n = 22) to explain the findings. Each interview was between 30 min and 1 hr and all interviewers were trained in realist interviewing techniques; interviews were audio recorded and transcribed verbatim. All data were analysed using a realist logic of analysis to make sense of, test and refine the programme theories (Manzano, 2016; Punton, Vogel, Vogel, & Lloyd, 2016). Qualitative data were transcribed verbatim, imported into NVivo and analysed using a realist CMO lens. During the data collection and analysis, we moved iteratively between analysis of particular examples, refinement of programme theory, and application of abstract theory (Emmel, 2013; Shearn, Allmark, Piercy, & Hirst, 2017; Wong et al., 2015).

Participants were recruited via CA (March–October 2016); CA staff provided questionnaires for clients to complete post first consultation and approximately 4–6 weeks later, and referred contact details of clients who expressed an interest in being interviewed to the research team. Six weeks was chosen as the approximate time for follow-up, as the WEMWBS literature states that follow-up data can be collected 2 weeks after the first administration of the questionnaire (Stansfield, Collins, Collins, Timpson, & Whelan, 2013), while for the PSS an interval of up to 4–6 weeks is recommended prior to decreased validity (Eun-Hyun, 2012). Purposive sampling was used to ensure that all three groups were represented in interviews. Whilst all sample groups received the same service, sampling across different projects enabled us to reach different target groups. No age, gender, or welfare issues were considered when recruiting clients. Citizens Advice clients are recognised as a potentially difficult population to recruit to research, given the complex social circumstances they are experiencing (Farr, Cressey, Cressey, Milner, Abercrombie, & Jaynes, 2014). Therefore, no further sampling criteria were applied, in order to avoid restricting the numbers of eligible participants. No further participant details were taken, in order to limit the time required to complete the questionnaires and interviews, and to ensure participants felt the data they provided would be anonymous. Using a pre- and postdesign, PSS and WEMWEBS data were analysed applying paired t-tests using the statistical software R (R Core Team, 2018). As the focus of the work was theory driven and explanatory, no control group was used. Further details of the operationalisation of the project are provided in Table 2 and are available in the study protocol (Forster, Dalkin, Lhussier, Hodgson, & Carr, 2016).

Initial programme theories were developed through the literature (Phase 1) and interviews with CA staff (Phase 2). A bespoke data recording template was then developed (Phase 3) and programme theories were refined and tested iteratively with the quantitative data and through client interviews (Phase 4). Typically, a realist analytical process includes to-ing and fro-ing between abstract theories and data, retroductively, in order to enable the development of best-fit explanatory programme theories. Given the realist approach taken, often concepts (such as stress) can feature differently within

TABLE 2 Operationalisation of the realist evaluation in five phases

Project phase	Methods
1. Building programme theories	Literature<br>Interviews with Citizens Advice Gateshead staff (*n* = 3)
2. Refining programme theories	Presentation of initial programme theories to staff for comment (*n* = 5)<br>Further interviews with Citizens Advice Gateshead staff (*n* = 3)
3. Development of a bespoke data recording template to capture long term impact	Collaborative work with Citizens Advice Gateshead staff
4. Testing programme theories with empirical data	Quantitative (questionnaire, *n* = 191, 91% follow up 4 weeks–6 months):<br>• Perceived stress scale [22]<br>• Warwick Edinburgh Mental Wellbeing Scale [23]<br>• Lifestyle questions<br>Qualitative:<br>• Realist interviews (Manzano, 2016) with Citizens Advice Gateshead clients (*n* = 22). Young persons' service *n* = 5; GP referral service *n* = 6; Mental health service *n* = 11.
5. Impact interviews	Interviews with staff concerning feasibility of ongoing use of questionnaires (*n* = 5)

individual CMOC, for example, being in one instance an outcome and in another, a mechanism (Pawson & Tilley, 1997).

The findings section below is structured using three overarching substantive theories that help to explain and understand the data at the middle range, and their component programme theories, with supporting data.

The reporting of this research study adheres to the Realist And Meta-narrative Evidence Syntheses: Evolving Standards (RAMESES) II guidelines for realist evaluation (Wong et al., 2016) (Supporting Information Table S1). Thus, in line with a realist approach, substantive theory is mixed with programme theory in the results section to enhance the explanatory endeavour of the study (Wong et al., 2016). Ethical approval was granted by the University Research Ethics Committee on 01/06/2015.

3 | RESULTS

3.1 | Quantitative data analysis

The analysis of the WEMWBS data indicated a significant difference between initial consultation and follow-up, with all included clients indicating an increase in well-being post advice ($p < 0.001$; 95% CI) (Figure 1); the mean scores were 26.9 on entry and 46.5 after advice. PSS analysis showed a uniformly downward trend, indicating that all clients were less stressed on their second visit ($p < 0.001$; 95% CI) with the initial mean of 31.4 and a post advice mean of 10.3 (Figure 2). Thus, quantitative findings demonstrate that clients experienced a reduction in stress following the receipt of advice. The qualitative data were used to understand the distinct contribution of CA to this outcome, the mechanisms through which this was achieved and the essential contexts which impacted upon this.

3.2 | Qualitative theory testing

In the following section, a brief overview of the findings is presented, first explaining the relevant substantive theory and detailing the refined programme theories relating to it. Specific initial programme theories are not detailed due to space limitations and the focus on the overall findings of the research, but are available upon request. What is presented is therefore the end product of the analysis as opposed to the unfolding story of theory testing. The overall initial programme theory tested was that, in the context of austerity, CA provides advice (resource) which reduces clients' stress (reasoning) leading to an increase in well-being (outcome).

3.2.1 | Overarching theory 1: The capabilities model

Sen's (1985, 1999, 1985, 1999, 2004) Capabilities Model provides a theoretical framework which best incorporates the social and political contexts of CA clients. It reflects well-being and quality of life within the boundaries of what a person is able to achieve, rather than using a standardised set of outcomes (such as income or desire fulfilment). In all the programme theories below, the advice provided by CA changes the set of capabilities that clients have, therefore allowing them to, for example, decrease social isolation.

Programme theory (PT) 1: Providing a stop gap

In a context where someone's basic needs are unmet (first acute issue is presented to CA), the provision of a stopgap (e.g. food bank voucher) or prevention strategy (e.g. prevention of homelessness) (resource) increases the individual's capabilities to meet their fundamental needs, leading to the person feeling relieved (reasoning), and resulting in a reduction in stress (outcomes).

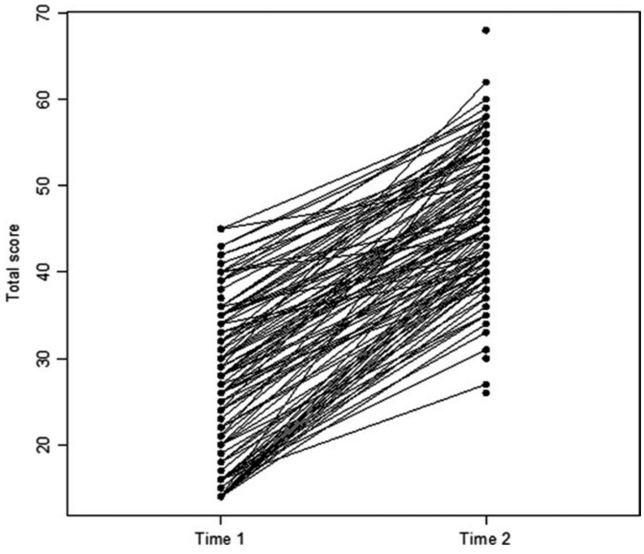

FIGURE 1 Clients' WEBWBS scores at Time 1 (initial consultation) and Time 2 (follow up)

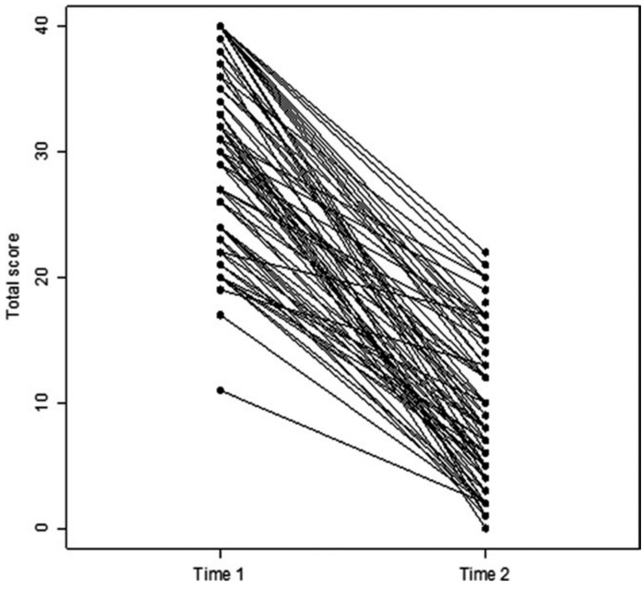

FIGURE 2 Clients' PSS scores at Time 1 (initial consultation) and Time 2 (follow up)

> so they helped me get er the benefits that, the benefit that I'm entitled to …like I says er, if I, I wouldn't of known anything, I would of ended up losing my flat …I would of ended up homeless and everything. Client 22

PT2: Taking control

During times of crisis, where stress is high and people feel out of control (context), CA staff take responsibility for finding solutions to people's issues (e.g. financial, employment, or housing issues) (resource), resulting in the person feeling less stressed and having greater emotional capability to deal with life circumstances (reasoning), leading to increased well-being (outcome).

PT3: Enabling social contact

(a) Where people are generally well but socially isolated (context), increased finances as a result of accessing CA (resource) increases their capabilities to engage in activities, leading the client to feel less socially isolated (reasoning) and experience an increase in well-being (outcome).

> erm well it's went from counting our pennies, well at one stage I had no money coming in and my husband had to support me, buy the food for the house, the electric, the gas everything… but we'd always been savers and the idea that I had to rely on him to lending me some money every week just so I had some money in me purse was… but you know as I say it's (CA) just helped so much it was unbelievable. I feel as if I've got part of me freedom back again. Client 16

(b) However, when someone has a severe, longstanding health issue (context), additional finances as a result of accessing CA (resource) impact less on clients' feelings of social isolation (reasoning) as their capabilities may remain unchanged due to their illness. Well-being may therefore remain the same (outcome).

PT4: Self-care for mental health

Stress is exacerbating a client's long-term mental health condition, which was previously under control (context). CA provides advice on the client's issue (resource) which reduces stress (reasoning) and allows them to engage in better self-care. Due to this reduction, the client is prevented from continuing to decline in their condition and can continue to self-manage (outcome).

In summary, through the provision of a range of resources, depending on individual circumstances (e.g. providing a stopgap or enabling access to additional income), CA reduce client stress and increase the capabilities of clients through enhancing the range of options available to them.

3.2.2 | Overarching theory 2: Decision to trust

Hurley (2006) describes decisions to trust in terms of the particular circumstance and relationship between "the truster" (in this study, the client) and trustee (CA staff). Hurley (2006) states that there are seven factors which impact on trust development: security, number of similarities, alignment of interests, benevolent concerns, capability, predictability and integrity, and level of communication. Two programme theories are underpinned by this model of trust:

PT5: Increased trust

In a context of anxiety generally about having a problem and from being let down by other services/not trusting other services, clients turn to CA as an alternative. CA staff demonstrates qualities that

meet the components of Hurley's trust model (similarities, alignment of interests etc.) (resource). This increases clients' trust in their named CA staff member (reasoning). Clients experience increased well-being and reduced anxiety (outcome) as a result of (a) increased hope that the problem will be resolved, (b) being able to access CA for future problems, and (c) feeling that somebody genuinely cares about them.

> yes 110% yes, I would trust her with anything. I felt comfortable with her she's, it was the way she spoke to you the way she like sort of stuck up for me there she she done an amazing job and she should be, there should be more like her really she's lovely. Client 11

PT6: Stigmatised position

People feel stigmatised by other organisations such as the Department of Work and Pensions (DWP) or the Jobcentre, by their peers (including family members), and wider society (context). CA provide nonjudgemental, personalised advice and normalise the process of claiming benefits (resource), thus adhering to several of the components of trust outlined by Hurley (Hurley, 2006); in particular the similarities dimension of the Decision to Trust model helped clients to feel a reduction in stigma (reasoning). The client feels less stigmatised and therefore validated in their needs and as people (reasoning). Well-being is increased through self-worth (outcome).

> I feel supported, like, there's people there to help you. See I felt last year, like, when I had to sign on to benefits, that people would judge. But then my partner had worked from being 16 and he was 25 so he'd never ever claimed benefits. He was embarrassed to go to the job centre. It was like a pride thing really. But because I'm a carer I was able to claim income support so he didn't have to go and feel embarrassed. Client 1

3.2.3 | Overarching theory 3: Third space theory

The concept of a Third Space (Bhabha, 2004) has been used in postcolonial literature to define culture as being located between two oppositional groups. It was used here to facilitate an understanding of the perceived incompatibility between state systems and the capabilities of CA clients. Without an intervening service, this incompatibility was felt to act as a barrier to positive outcomes. However, by creating a flexible Third Space, CA was able to achieve positive outcomes, such as reduced stress, by identifying underlying issues for clients and interpret them into state systems. The first theory below (CA as a Buffer) describes how this operates in practice, with the other two programme theories (Form filling, Tribunal Attendance) providing more practical accounts of how the buffer is created.

PT7: CA acting as a buffer

In the context of a distrust of the state (context) CA acts as a person of standing offering effective, impartial and non-judgemental services (resource) which allow people to feel supported and to develop trust (reasoning). This results in CA creating a "Third Space" or a buffer between the person and the state (outcome 1), which results in a decrease in stress (outcome 2) and higher likelihood of access to benefits (outcome 3).

> it is a worry if the citizens advice is not there I think people like me wouldn't have anybody to turn to, the government would tell us what's what and that would be it and er it's a case of know your place and do as you're told. Client 5

PT8: Form filling

The client is stressed and may have literacy or mental health issues (context). CA acts as an expert system navigator filling in forms for clients using their knowledge of the system (resource), thus allowing the client and state to interact more efficiently. The client is relieved and has increased trust due to CA staff knowledge and consistent support (reasoning). Stress decreases and the form is processed successfully (outcome).

> well these people here (at CA), help you fill the forms out, and understand, and help you put in it, and they put in it about the mental and the physical. Because they understand, rule this and section that and, which the normal person in the street isn't going to have a bloody clue about. And even if you try to look it up online, you look stuff up online, you just go woah, what's that about? Client 9

PT9: Tribunal attendance

The client is requested to attend an appeal and is stressed (context). CA prepare for and attend the appeal on behalf of, or with the client and offer an informed expert opening statement (resource), thus conveying information to the state on the client's behalf in an effective way. The client trusts CA expertise, making them feel more comfortable and reassured (reasoning). The client is less stressed and maintains engagement with the process. This also reinforces CA's position as an expert system navigator (outcome).

The use of abstract theory in combination with programme theories has allowed investigation at several layers of the system—the individual (Capabilities model), the interaction between the individual and the CA staff (Decision to Trust model) and the interaction between the client, CA and the state (Third Space).

4 | DISCUSSION

The programme theories developed and tested in this study focused on explaining how, why and in which circumstances intensive advice

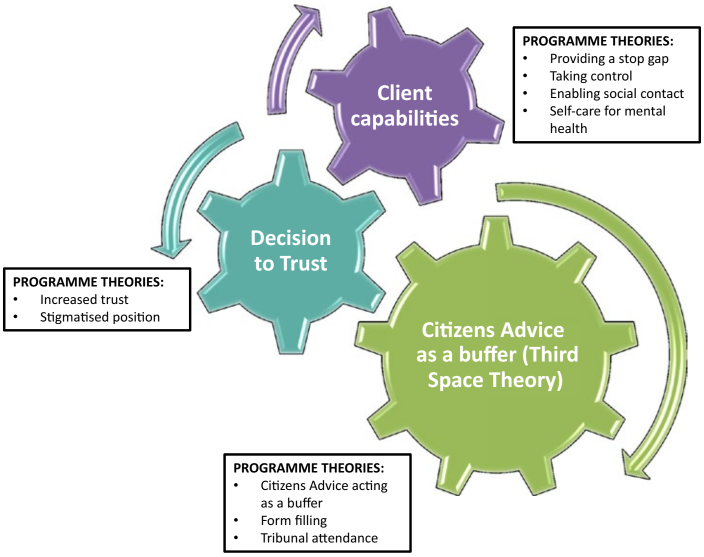

FIGURE 3 Conceptual representation of the ways in which CAG impacts clients' stress and wellbeing

services had an impact on stress and well-being. The theories were refined and tested using both substantive theories and empirical data. The findings suggest that CA impact on well-being and stress through (a) increasing clients capabilities (b) fostering a trusting relationship and (c) by creating a facilitative Third Space to act as a buffer between the oppositional positions of the client and the state (Figure 3). Figure 3 shows the relationship between the abstract and programme theories and highlights how better outcomes are achieved for clients when all "cogs" are in action. The figure provides an explanatory framework, which accounts for the different levels through which CA improve people's mental health (which may therefore impact physical health); client capabilities representing the micro, the meso being the trusting relationship between the client and the CA advisor, and the macro being the buffering role CA form between clients and the state. Previous realist research has drawn on substantive theories focused at macro, meso, and micro levels at the outset of the research in order to support the development of more specific program theories (Shearn et al., 2017). By contrast, our research built theory in the opposite direction, from programme theories anchored in the data to the construction of an abstract explanatory framework operating at these different layers.

Study findings contribute understanding around the potential for public services and institutions to perpetuate or mitigate vulnerability. Recent work argues for relational approaches that understand vulnerability not only by reference to the state and institutional conditions that produce it, but the agency and capacities of individuals as they negotiate access to required resources amid these broader social and political constraints (Emmel, 2017). The multilayered nature of the explanatory framework developed, which is both empirically and theoretically substantiated, illustrates the relationship between the resources and capabilities of CA clients, and the broader socio-political climate. Crucially, it highlights the importance of the mediating role of CA services, understood here as the creation of a facilitative Third Space between two opposed groups, where clients may themselves lack agency in engaging directly with the state and the impact this has in reducing stress and improving well-being. In doing so, this paper also responds to calls for greater research on the "empirical realities" of vulnerability (Brown, Ecclestone, Ecclestone, & Emmel, 2017) from the perspectives of both advice service providers and clients.

The findings from this research support existing literature highlighting the impact of advice services on mental health and well-being (Abbott, 2002; Burrows et al., 2011; Citizens Advice Bureau, 2012; Holkar & Mackenzie, 2016), although the service evaluated here was an intensive service where clients had longer term contact, which may have led to slightly enhanced outcomes. Research has also highlighted the complexity of identifying links between advice services and health (Allmark et al., 2013). This research begins to map the pathways of impact from advice to reduced stress and increased well-being, which are recognised as improving more distal, long term health outcomes, such as cardiovascular disease (Dimsdale, 2008), diabetes control (Faulenbach et al., 2012), and rheumatoid arthritis (Straub, Dhabhar, Dhabhar, Bijlsma, & Cutolo, 2005). However, outcomes of this type were not demonstrable in the study and it is acknowledge that potentially reduction in stress and increase in well-being could lead to no further health effects.

Our research also builds upon previous research by evidencing the mechanisms and contexts within which these outcomes are achieved. The use of a stress lens in this field in particular was novel but initially debated amongst the research and practice teams. Clients who accessed the projects did so with individualised issues; it was therefore difficult to identify a set of outcomes which could relate to all clients without taking a generalised lens, using stress and well-being. Debates occurred surrounding the relative value of including topic specific outcome measures (e.g.

amount of debt or housing status) that could then be related to health using the literature. Many of these outcome measures are already recorded by CA. Furthermore, given the individual character and complexity of clients' circumstances proximal outcomes such as stress and well-being could apply to all clients, therefore providing more power for statistical analysis. Long term, PSS, and WEMWBS could allow insights into health outcomes despite clients' diverse circumstances. Furthermore, measures of increased finances as a result of accessing CA would never be sufficient to take clients from a position of socioeconomic deprivation to socioeconomic stability and this therefore can make it difficult for clients to change their health behaviours (Venn & Strazdins, 2017); this is a limitation of the welfare regime in and of itself.

One further challenge related to timing of the administration of the follow -up questionnaire. Whilst we aimed to collect data 4–6 weeks post initial contact, access issues meant that follow-up data were collected between 6 weeks and 6 months. Within this wider period reliability of the PSS is decreased and there is a higher likelihood that other factors independent of the programme could have also attributed to stress and well-being outcomes. However, many clients received advice over a long period of time (up to 2 years) and interviews suggested that stress reduction and increased well-being were attributable to the programme. The wider data collection period also meant that some clients had received an outcome in relation to the issue they approached CA with, and others had not. However, it is notable that stress had remained decreased and well-being increased despite this lengthier follow-up period and varying outcome status. Furthermore, interview data indicated that clients were mainly still positive about CA, even where outcomes had not been in their favour, due to acknowledgement of the effort the service had made to help them address their issues.

There could be expectations of bias in participants' responses when completing the questionnaires, as this was sometimes completed whilst the CA advisor was present. However, the questionnaires were about the participants stress and well-being, not about the service they had received, therefore answers provided could be given honestly, as it was a reflection of their emotional state as opposed to the service or the CA advisor.

The short-term outcomes of this study could also be seen as a limitation, however, the links between stress, well-being, and more physical health have been clearly demonstrated in the literature. However, the causal pathway from advice to increased well-being and decreased stress are not clearly understood and therefore comprised the focus of this study.

5 | CONCLUSION

In a UK context of austerity, funders are increasingly under pressure to commission services which are well-evidenced to impact on health; this can be supported by better data collection from advice services (Abbott & Hobby, 2003). This research, which was conducted in close collaboration with CA staff, has generated insights for commissioners, advice services, and researchers around the health outcomes of advice services and how these can best be assessed. The study not only identifies positive outcomes for clients as a result of accessing advice services, but highlights the contexts and mechanisms through which this occurs. Use of a stress and well-being lens has provided more generalised findings and allowed insight into the precursors of health. When paired with an understanding of contextual and mechanistic properties, this begins to build a more complex and real picture of how advice services impact on health.

This study also has important practice and policy implications. In terms of practice, longer term contact with clients may result in better outcomes through increased trust. It could be suggested that commissioning should look to protect this model of service delivery, particularly with those experiencing multiple and complex issues. Policy makers need to be aware of adversarial systems which prevent clients from accessing entitlements. In this circumstance, CA provides essential help to allow the state and clients to interact effectively.

The use of realist evaluation as a theory-informed approach, has enabled the production of insights which have transferability across contexts. Future research should further this understanding by testing the explanatory framework generated with a larger sample, or in other welfare services.

ACKNOWLEDGEMENTS

The authors acknowledge our practice partners Citizens Advice Gateshead and thank all the participants who took part in the study. We also thank Fuse (The Centre for Translational Research in Public Health) for their support throughout the project.

CONFLICT OF INTEREST

No conflicts of interest have been declared.

ORCID

Sonia Michelle Dalkin https://orcid.org/0000-0002-3266-5926

REFERENCES

Abbott, S. (2002). Prescribing welfare benefits advice in primary care: Is it a health intervention, and if so, what sort? *Journal of Public Health*, *24*(4), 307–312. https://doi.org/10.1093/pubmed/24.4.307

Abbott, S., & Hobby, L. (2003). Who uses welfare benefits advice services in primary care? *Health and Social Care in the Community*, *11*(2), 168–174. https://doi.org/10.1046/j.1365-2524.2003.00414.x

Abbott, S., Hobby, L., & Cotter, S. (2005). What is the impact on individual health of services in general practice settings which offer welfare benefits advice? *Health and Social Care in the Community*, *14*(1), 1–8. https://doi.org/10.1111/j.1365-2524.2005.00582.x

Acheson, D. (1988). *Independent inquiry into inequalities in health*. London: HMSO.

Adams, J., White, M., Moffatt, S., Howel, D., & Mackintosh, J. (2006). A systematic review of the health, social and financial impacts of

welfare rights advice delivered in health care settings. *BMC Public Health*, *6*(1), 81. https://doi.org/10.1186/1471-2458-6-81

Allmark, P., Baxter, S., Goyder, E., Guillaume, L., & Crofton-Martin, G. (2013). Assessing the health benefits of advice services: Using research evidence and logic model methods to explore complex pathways. *Health and Social Care in the Community*, *21*(1), 59–68. https://doi.org/10.1111/j.1365-2524.2012.01087.x

Benzeval, M., Bond, L., Campbell, M., Egan, M., Lorenc, T., Petticrew, M., & Popham, F. (2014). *How Does Money Influence Health?* York: Joseph Rowntree Foundation.

Bhabha, H. (2004). *The Location of Culture*. New York: Psychology Press.

Black, D., Morris, J. N., Smith, C., & Townsend, P. (1980). *Black Report. Inequalities in health: Report of a research working group*. London: Department of Health and Social Security.

Bridges, S. (2014). Chapter 2: Mental health problems. Health Survey for England, Vol. 1. Retrieved from https://digital.nhs.uk/dataandinformation/publications/statistical/health-survey-for-england/health-survey-for-england-2014

Brown, K., Ecclestone, K., & Emmel, N. (2017). The many faces of vulnerability. *Social Policy and Society*, *16*(3), 497–510. https://doi.org/10.1017/S1474746416000610

Burrows, J., Baxter, S., Baird, J., Hirst, E., & Goyder, E. (2011). Citizens advice in primary care: A qualitative study of the views and experiences of service users and staff. *Public Health*, *125*, 704–710.

Citizens Advice Bureau. (2012). *An Overview of Possible Links Between Advice and Health*. London: Citizens Advice Bureau.

Cohen, S., Kamarck, T., & Mermelstein, R. (1983). A global measure of perceived stress. *Journal of Health and Social Behavior*, *24*(4), 385.

Dalkin, S., Greenhalgh, G., Jones, D., Cunningham, B., & Lhussier, M. (2015). What's in a mechanism? Development of a key concept in realist evaluation. *Implementation Science*, *10*, 49. https://doi.org/10.1186/s13012-015-0237-x

Dalkin, S., Jones, D., Lhussier, M., & Cunningham, W. (2012). Understanding integrated care pathways in palliative care using realist evaluation: A mixed-methods study protocol. *British Medical Journal Open*, *2*(4), pii: e001533. https://doi.org/10.1136/bmjopen-2012-001533.

Dalkin, S. M., Lhussier, M., Philipson, P., Jones, D., & Cunningham, W. (2016). Reducing inequalities in care for patients with non-malignant diseases: Insights from a realist evaluation of an integrated palliative care pathway. *Palliative Medicine*, *30*(7), 690–697. https://doi.org/10.1177/0269216315626352

Dalkin, S., Lhussier, M., Williams, L., Burton, C., & Rycroft-Malone, J. (2018). Exploring the use of Soft Systems Methodology with realist approaches: A novel way to map programme complexity and develop and refine programme theory? *Evaluation*, *24*(1), 84–97. https://doi.org/10.1177/1356389017749036

Dimsdale, J. (2008). Psychological stress and cardiovascular disease. *Journal of the American College of Cardiology*, *51*(13), 1237–1246. https://doi.org/10.1016/j.jacc.2007.12.024

Edwards, P., Jarvis, J., Shaw, K., & Irving, A. (2013). The Impact of Welfare Reform in the North East. A research report for the Association of North East Councils by the Universities of Durham (Institute for Local Governance), Northumbria and Teesside and the North East region of Citizens Advice.

Emmel, N. (2013). *Sampling and choosing cases in qualitative research: A realist approach*. London: Sage.

Emmel, N. (2017). Empowerment in the relational longitudinal space of vulnerability. *Social Policy and Society*, *16*(3), 457–467. https://doi.org/10.1017/S1474746417000021

Eun-Hyun, L. (2012). Review of the psychometric evidence of the perceived stress scale. *Asian Nursing Research*, *7*(3), 160.

Farr, M., Cressey, P., Milner, S., Abercrombie, N., & Jaynes, B. (2014). *Proving the value of advice: A study of the impact of Citizens' Advice Bureau services*. Bath: University of Bath.

Faulenbach, M., Uthoff, H., Schwegler, K., Spinas, G., Schmid, C., & Wiesli, P. (2012). Effect of psychological stress on glucose control in patients with Type 2 diabetes. *Diabetic Medicine*, *29*(1), 128–131.

Forster, N., Dalkin, S., Lhussier, M., Hodgson, P., & Carr, S. (2016). Exposing the impact of advice services on health and inequalities: A realist evaluation protocol. *British Medical Journal Open*;*6*(1):e009887.

Gianaros, P., & Wager, T. (2015). Brain-body pathways linking psychological stress and physical health. *Current Directions in Psychological Science*, *24*(4), 313–321. https://doi.org/10.1177/0963721415581476

Greenhalgh, J., Dalkin, S., Gooding, K., Gibbons, E., Wright, J., Meads, D., … Pawson, R. (2017). Functionality and feedback: A realist synthesis of the collation, interpretation and utilisation of patient-reported outcome measures data to improve patient care. *NIHR Health Services and Delivery Research*, *5*(2), 1–280.

Hacking, J., Muller, S., & Buchan, I. (2011). Trends in mortality from 1965 to 2008 across the English north-south divide: Comparative observational study. *BMJ*, *342*, d508–d508. https://doi.org/10.1136/bmj.d508

Hirst, J., & Minter, S. (2014). *Citizens Advice Bureaux in General Practice Report 2013/14*. Derbyshire: Derbyshire County Council and Citizens Advice Bureau.

Holkar, M., & Mackenzie, P. (2016). *Money on your mind*. London: Money and Mental Health Policy Institute.

Hurley, R. (2006). The decision to trust. *Harvard Business Review*, *84*(9), 55–62.

Manzano, A. (2016). The craft of interviewing in realist evaluation. *Evaluation*, *22*(3), 342–360. https://doi.org/10.1177/1356389016638615

Marmot, M. (2010). *Fair society, healthy lives: The Marmot Review: Strategic review of health inequalities in England post-2010*. London: University College London.

Mental Health Foundation (2016). *Fundamental facts about mental health 2016*. London: Mental Health Foundation.

Moffatt, S., Lawson, S., Patterson, R., Holding, E., Dennison, A., Sowden, S., & Brown, J. (2016). A qualitative study of the impact of the UK 'bedroom tax'. *Journal of Public Health*, *38*(2), 197–205.

Moffatt, S., & Scambler, G. (2008). Can welfare-rights advice targeted at older people reduce social exclusion? *Ageing and Society*, *6*, 875–899. https://doi.org/10.1017/S0144686X08007253

Office for National Statistics (2014). *Healthy Life Expectancy at birth for Upper Tier Local Authorities: England, 2010–12*. London: Office for National Statistics.

Office for National Statistics (2015). *Suicides in the United Kingdom, 2013 Registrations*. London: Office for National Statistics.

Pawson, R., & Tilley, N. (1997). *Realistic evaluation*. London: Sage.

Punton, M., Vogel, I., & Lloyd, R. (2016). Reflections from a realist evaluation in progress: Scaling ladders and stitching theory. Centre for Development Impact.

R Core Team. (2018). *R: A language and environment for statistical computing*. Retrieved from https://www.R-project.org/

Sen, A. (1985). *Commodities and capabilities*. Amsterdam: North-Holland.

Sen, A. (1999). *Development as freedom*. Oxford: Oxford University Press.

Sen, A. (2004). Capabilities, lists, and public reason: Continuing the conversation. *Feminist Economics*, *10*(3), 77–80. https://doi.org/10.1080/1354570042000315163

Shearn, K., Allmark, P., Piercy, H., & Hirst, J. (2017). Building realist program theory for large complex and messy interventions. *The International Journal of Qualitative Methods*, *16*, 1–11.

Stansfield, J., Collins, B., Timpson, H., & Whelan, G. (2013). *Using and analysing WEMWBS to measure the impact of interventions in improving mental wellbeing*. Liverpool John Moores University, Champs Public Health Collaborative. Retrieved from http://www.champspublichealth.com/sites/default/files/event_files/WEMWBS%20workshop%20workbook%20FINAL.pdf

Straub, R., Dhabhar, F., Bijlsma, J., & Cutolo, M. (2005). How psychological stress via hormones and nerve fibers may exacerbate rheumatoid

arthritis. *Arthritis and Rheumatology*, *52*(1), 16–26. https://doi.org/10.1002/art.20747

Tennant, R., Hiller, L., Fishwick, R., Platt, S., Joseph, S., Weich, S., … Stewart-Brown, S. (2007). The Warwick-Edinburgh Mental Well-being Scale (WEMWBS): Development and UK validation. *Health and Quality of Life*, *5*, 63. https://doi.org/10.1186/1477-7525-5-63

Venn, D., & Strazdins, L. (2017). Your money or your time? How both types of scarcity matter to physical activity and healthy eating. *Social Science & Medicine*, *172*, 98–106.

Welfare Reform Act. (2012). Retrieved from http://www.legislation.gov.uk/ukpga/2012/5/contents/enacted

Whitehead, M. (2014). *Due North: Report of the Inquiry on Health Equity for the North*. Great Britain: University of Liverpool and Centre for Local Economic Strategies.

Wong, G., Brennan, N., Mattick, K., Pearson, M., Briscoe, S., & Papoutsi, C. (2015). Interventions to improve antimicrobial prescribing of doctors in training: The IMPACT (IMProving Antimicrobial presCribing of doctors in Training) realist review. *British Medical Journal Open*, *5*(10), e009059.

Wong, G., Westhorp, G., Manzano, A., Greenhalgh, J., Jagosh, J., & Greenhalgh, T. (2016). RAMESES II reporting standards for realist evaluations. *BMC Medicine*, *14*(1), 96. https://doi.org/10.1186/s12916-016-0643-1

Woodhead, C., Khondoker, M., Lomas, R., & Raine, R. (2017). Impact of co-located welfare advice in healthcare settings: Prospective quasi-experimental controlled study. *The British Journal of Psychiatry*, *211*(6), 388–395. https://doi.org/10.1192/bjp.bp.117.202713

SUPPORTING INFORMATION

Additional supporting information may be found online in the Supporting Information section at the end of the article.

How to cite this article: Dalkin SM, Forster N, Hodgson P, Lhussier M, Philipson P, Carr SM. Exposing the impact of intensive advice services on health: A realist evaluation. *Health Soc Care Community*. 2018;00:1–10. https://doi.org/10.1111/hsc.12695
