# Supplementary material for: ‘Everything takes too long and nobody is listening’: Developing theory to understand the impact of advice on stress and the ability to cope
Source: PLoS One. 2020 Apr 23;15(4):e0231014. doi: 10.1371/journal.pone.0231014 (PMC7179918; doi:10.1371/journal.pone.0231014)
Supplement: S3 File — (DOCX) [file pone.0231014.s003.docx]

**Consolidated criteria for reporting qualitative research (COREQ)**

**Article: “*Everything takes too long and nobody is listening”:* Developing theory to understand the imapct of advice on stress and the ability to cope.**

| Personal Characteristics |  |  |
| --- | --- | --- |
| 1. | Interviewer/facilitator | SMD / PH / NF |
| 2. | Credentials | All PhD |
| 3. | Occupation | Lecturer in Public Health and Wellbeing / Senior Research Assistant / Senior Research Assistant |
| 4. | Gender | Female / Male / Female |
| 5. | Experience and training | All researchers had previously carried out qualitative research. All researchers used qualitative research as part of the PhD. |
| Relationship with participants |  |  |
| 6. | Relationship established | No |
| 7. | Participant knowledge of the interviewer | Participants knew the researchers place of work and that they were conducting research into one of the services provided by Citizens Advice. |
| 8. | Interviewer characteristics | None. |
| **Domain 2: study design** |  |  |
| Theoretical framework |  |  |
| 9. | Methodological orientation and Theory | Constructionist thematic analysis |
| Participant selection |  |  |
| 10. | Sampling | Purposive |
| 11. | Method of approach | Telephone |
| 12. | Sample size | 22 |
| 13. | Non-participation | Unknown |
| Setting |  |  |
| 14. | Setting of data collection | Participants home or at their local Citizens Advice |
| 15. | Presence of non-participants | No |
| 16. | Description of sample | All participants had been referred to one of Citizens Advice’s intensive advice service, for those with complex and multiple issues. Participants were between 16 and 70 years old. |
| Data collection |  |  |
| 17. | Interview guide | An interview schedule was used by the authors. This was not piloted but was checked by the project team and steering group. |
| 18. | Repeat interviews | No |
| 19. | Audio/visual recording | Audio recording |
| 20. | Field notes | Yes |
| 21. | Duration | 30-60 minutes |
| 22. | Data saturation | Yes |
| 23. | Transcripts returned | No |
| **Domain 3: analysis and findings** |  |  |
| Data analysis |  |  |
| 24. | Number of data coders | 3 (SMD, PH, NF) |
| 25. | Description of the coding tree | Yes, in the form of themes and sub themes. |
| 26. | Derivation of themes | Derived from the data |
| 27. | Software | QSR NVivo |
| 28. | Participant checking | No. |
| Reporting |  |  |
| 29. | Quotations presented | Quotations presented and participant identified. |
| 30. | Data and findings consistent | Yes |
| 31. | Clarity of major themes | Yes |
| 32. | Clarity of minor themes | Yes |
